# Stunting Reversal versus Regression to the Mean in Epidemiological Studies of Child Linear Growth in Low- and Middle-Income Countries^☆^

**DOI:** 10.1016/j.advnut.2026.100688

**Published:** 2026-06-20

**Authors:** Daniel E Roth, Kelly M Watson, Huma Qamar, Diego G Bassani

**Affiliations:** 1 Centre for Global Child Health, The Hospital for Sick Children, Toronto, ON, Canada; 2 Department of Paediatrics, University of Toronto, Toronto, ON, Canada; 3 Dalla Lana School of Public Health, University of Toronto, Toronto, ON, Canada; 4 Department of Nutritional Sciences, University of Toronto, Toronto, ON, Canada; 5 Graduate Entry Medical Programme, University of Limerick, Limerick, Ireland

**Keywords:** linear growth faltering, height-for-age *z*-scores, stunting, child growth, low- and middle-income countries, regression to the mean, mathematical coupling, epidemiology

## Abstract

Early childhood undernutrition in low- and middle-income countries (LMICs) is marked by population-wide linear growth faltering, whereby most children grow in height more slowly than expected for their age and a relatively high proportion of children are classified as stunted based on a height-for-age *z*-score (HAZ) below −2. In epidemiological studies in LMICs, patterns of linear growth are often examined by longitudinal analyses of individual children’s HAZ trajectories by age; in some studies, catch-up growth is defined as an upward HAZ threshold-crossing event such as recovery from stunting or stunting reversal (i.e., crossing from below to above HAZ = −2). In this Perspective, we question the suitability of this method of studying catch-up growth and highlight its susceptibility to regression to the mean (RTM), a statistical phenomenon whereby individuals with extreme values tend to move closer to the population mean at subsequent measurement timepoints. We use simulations of anthropometric datasets to demonstrate that upward HAZ threshold-crossing events (e.g., stunting reversal) are caused by RTM, and we discuss the advantages and pitfalls of other statistical methods to address RTM. In a scoping review of epidemiological studies of linear growth catch-up in LMICs (2004 to mid-2024; *N* = 69), we found that RTM was uncommonly mentioned (20%), and few studies (5/69; 7%) explicitly addressed RTM. In most studies (36/69; 52%), inferences about catch-up were based on methods that are at higher risk of RTM artifacts, namely by quantifying threshold-crossing events (*n* = 24) or by stratification of the sample by baseline size (*n* = 12). We conclude that more attention to RTM is needed in analyses of linear catch-up growth in LMICs. Published studies that used RTM artifact-prone measures should be remediated, and the use of HAZ threshold-crossing events such as stunting reversal should be avoided in future research.

## Introduction

**Statement of significance**

Regression to the mean (RTM) is a statistical phenomenon that has been largely ignored or misinterpreted in epidemiological studies of linear growth catch-up patterns in low- and middle-income countries. We call attention to the pervasiveness of this problem in the published literature, discuss methods that account for RTM, and caution against the use of metrics such as stunting reversal and recovery, which are particularly prone to RTM artifacts.

In low- and middle-income countries (LMICs), children tend to grow more slowly than expected for their chronological age [1]. This phenomenon, known as linear growth faltering, is commonly expressed using sex- and age-standardized *z*-scores of children’s lengths or heights based on the WHO child growth standards (WHO-GS) [2]. In most LMICs, the population height-for-age *z*-score (HAZ) distribution is shifted down, such that its mean HAZ is negative and a relatively high proportion of children have heights (or lengths) below the threshold used to define stunting (HAZ = −2) [3]. The prevalence of stunting (percentage of children under 5 y of age with HAZ < −2) is a core public health indicator of nutritional status used for monitoring and tracking progress toward global targets [4].

Catch-up growth of children in LMICs has been defined and analyzed using a wide array of methods [[5], [6], [7], [8], [9], [10]]. The term catch-up should refer to a period of relatively accelerated growth that constitutes at least partial recovery following a period of growth restriction [9,10]; however, there is debate about the measurement scale on which an individual child’s or a population’s average catch-up growth rate is most appropriately measured [7,8]. Leroy et al. [7,11] have proposed that catch-up should only be measured using changes in absolute height (i.e. measured in centimeters) compared with the WHO-GS median, expressed as the height-for-age difference (HAD) rather than HAZ. However, HAD and HAZ conventionally entail comparisons of a child or population to the WHO-GS based on chronological age without consideration of skeletal maturation; this approach is inconsistent with the theory of delayed growth plate senescence, a biological mechanism that explains faltering and catch-up [12,13]. Therefore, for population-level analyses, we previously proposed an alternative approach using height-age (the age at which a population’s mean height equals the WHO-GS median, applied as a proxy for average skeletal maturity) and growth delay (the difference between the population’s chronological age and height-age) [8,14]. Using these metrics, a population is considered to be catching-up when its observed mean height tracks along the WHO-GS median for height-age, such that there is no further accrual of growth delay [8].

Anthropometric *z*-score classifiers such as stunting are defined by arbitrary statistical thresholds and lack clinical significance for individual children [15,16], yet individual children are often classified in epidemiologic studies as stunted (or not), perhaps motivated by a view of stunting as a disease or clinical syndrome [17]. This practice has led researchers to further label children who are stunted at one age and nonstunted at a subsequent age as a special group experiencing a form of catch-up referred to as stunting reversal or recovery from stunting [5,18]. Although the prevalence of stunting is an acceptable statistical indicator of the cross-sectional position of a population’s HAZ distribution relative to the WHO-GS at a given age or within a defined age range [14], population growth metrics based on the WHO-GS HAZ scale (i.e. mean HAZ, stunting prevalence, and incidence of stunting reversal) cannot be directly compared across different ages; for example, stunting prevalence may decline as a cohort of children ages even though the population continues to experience ongoing growth faltering [8,14,19].

Despite prior publications highlighting the pitfalls of using HAZ and stunting classifiers in epidemiologic research [7,8,20], these measures continue to be widely used, and stunting reversal has become a prominent metric of catch-up growth in the literature [18]. Therefore, in this Perspective, we take an alternative approach to critiquing methods that quantify catch-up growth on the HAZ scale by considering their susceptibility to regression to the mean (RTM), a statistical phenomenon whereby individuals with extreme values tend to move closer to the population mean at subsequent measurements [21]. The effects of RTM on HAZ threshold-crossing events are primarily of concern among researchers who use threshold-crossing metrics (and practitioners who rely on evidence from such studies). Although many of the arguments presented in this study may be seen as moot points among the (relatively few) researchers who have all but abandoned the use of HAZ when analyzing catch-up patterns, we caution that RTM is insidious, perplexing, and widely ignored. We therefore encourage all readers to consider this Perspective as a methodological case study to prompt attention to RTM in all linear growth analyses irrespective of the anthropometric measures, scales, or analytical methods used.

RTM in the context of studying BMI and obesity has been previously described by Thomas et al. [22]. In this study, we extend the discussion of RTM to observational studies that reported reversal of stunting or used other descriptions of catch-up growth of individual children in LMIC cohorts. We used simulations to create visualizations of RTM and conducted a scoping review of the relevant literature to demonstrate the extent to which RTM artifacts may have impacted the existing evidence base generated over the past 2 decades (Supplemental Methods). First, we address RTM in the context of longitudinal cohort studies in which individual children are repeatedly measured and catch-up is ascertained at the individual level; in that context, we focus primarily on RTM on the HAZ scale because of its widespread use in this field and because stunting classification (and hence, recovery from stunting) is contingent on the use of a HAZ threshold (−2) that defines stunting. Second, we consider RTM as it pertains to population-level trajectories; in that context, we revisit a widely cited methodological study of RTM and catch-up growth by Cameron et al., published in 2005 [23]. A brief note about terminology is warranted: we adopt terms used in published literature—recovery from stunting and stunting reversal—to indicate discrete upward HAZ threshold-crossing events (from below –2 to above –2), whereas catch-up growth is used to more generically refer to any upward trajectory on any measurement scale that might be interpreted as recovery following a period of faltering, without requiring that a specified HAZ or height threshold is crossed. Our permissive use of these terms reflects how catch-up phenomena have been reported and operationalized in the literature, without endorsing these definitions or operationalizations.

## Mechanisms and Consequences of RTM in Longitudinal Studies of Within-Child Changes in HAZ

**FIGURE 1:**
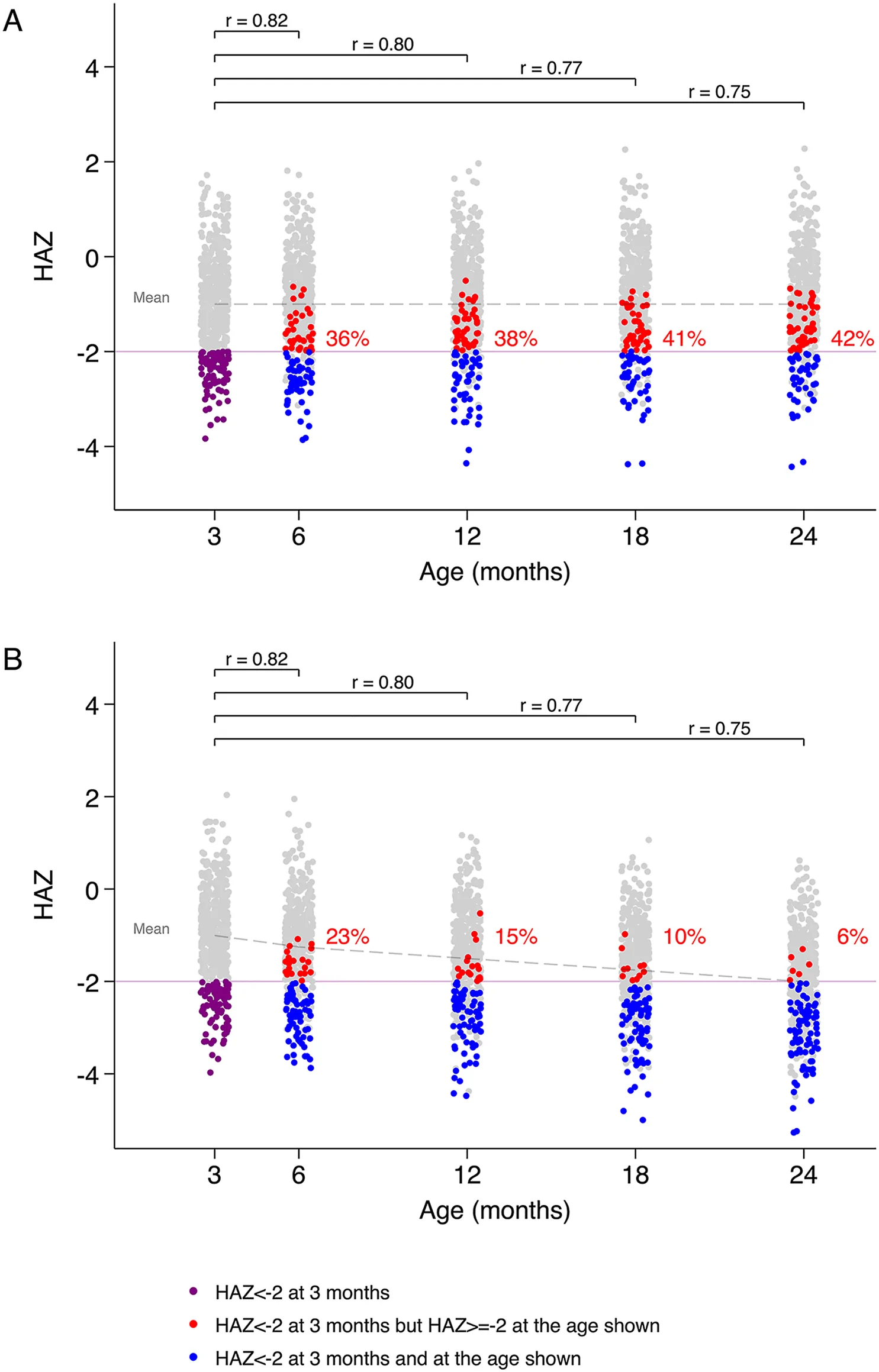
Threshold-crossing events [from below to above HAZ = −2] in a simulated longitudinal dataset of children (*N* = 100,000) followed from 3 to 24 mo of age with stable mean HAZ = −1 (A) or whole-population faltering pattern where mean HAZ declines by 0.25 at each successive age (B). Percentages denote the number of children with measurements above the HAZ = −2 threshold at the age (red dots) among those with HAZ < −2 at 3 mo (purple). All threshold-crossing events represent RTM due to the imperfect between-timepoint correlations (*r*) of repeated length measurements. The *r* values were based on a Bangladeshi birth cohort (See Supplemental Methods for details). Application of a conventional published formula to estimate the RTM effect [21] in the subgroup of those with HAZ < −2 at 3 mo gives an estimated change in the mean HAZ of +0.28 (from −2.52 at 3 mo to −2.25 at 6 mo) entirely due to RTM; this is correct for the stable mean HAZ scenario (A) but is misleading in the faltering scenario (B), in which the subgroup’s mean HAZ at 6 mo (−2.50) is relatively unchanged from 3 mo (−2.53) because the RTM effect (+0.28) is nearly offset by the downward whole-population shift (−0.25). The subgroup mean HAZ at 6 mo due to RTM is correctly predicted in both scenarios by the full conditional growth regression model (Equation *2*, in main text) but the simplified form (Equation *3*, in main text) yields an estimated mean HAZ at 6 mo of −2.07, which is incorrect for either scenario because it inappropriately assumes a stable mean HAZ = 0. A random 0.5% subsample of observations are used in the plots to aid in visualizing threshold-crossing event frequencies at each age. HAZ, height-for-age *z*-score; RTM, regression to the mean.

RTM occurs whenever a variable is measured in each individual more than once and the correlation between the 2 measurements is imperfect [24]. In longitudinal analyses of linear growth datasets (whereby each child is followed up over time and measured on ≥2 occasions), correlations between serial measures are always imperfect due to a combination of biological variability in the rates of linear growth (within-child variability) and technical errors of measurement (within-encounter variability) [25]. RTM is a prominent feature of the prototypical inverse correlation between baseline values and the changes between each baseline and its paired later measurement; the farther a baseline value is from the group mean, the larger the expected magnitude of change [24]. RTM should be assumed to affect any analysis of changes in HAZ in which children are grouped by, or selected on, baseline HAZ values (or characteristics that are associated with baseline HAZ). Children selected from a tail of the baseline HAZ distribution are expected to be positioned, on average, closer to the overall population mean at a follow-up timepoint; the magnitude of RTM is inversely proportional to the between-timepoint correlation of HAZ values (measured in the whole population from which the subgroup is drawn) [21]. The RTM effect is not expected to be consistent across the age range; for example, it is more pronounced at younger ages and with greater time spans between baseline and endline height measurements [26], as a longer time span allows a greater opportunity for biological variability in children’s growth (Figure 1) (See Supplemental Methods for details). In any population with mean HAZ above −2 at baseline, subsequent measurements of stunted children will tend to be closer to the population mean HAZ; thus, some of the stunted children will cross above the −2 threshold, giving rise to what has been labeled as stunting reversal, whether the the population has a stable HAZ distribution or is faltering relative to the WHO-GS (Figure 1). The magnitude of RTM and the frequency of threshold-crossing events may differ across otherwise similar studies due to numerous factors including procedures that influence the precision of anthropometric measurements [27,28]. In theory, RTM effects may be mitigated by efforts to reduce technical errors in anthropometry (e.g. training of data collectors, quality control, supervision, using repeated measurements, and calibration and standardization of scales) but will always be present when serial measurements are not perfectly correlated.

**FIGURE 2:**
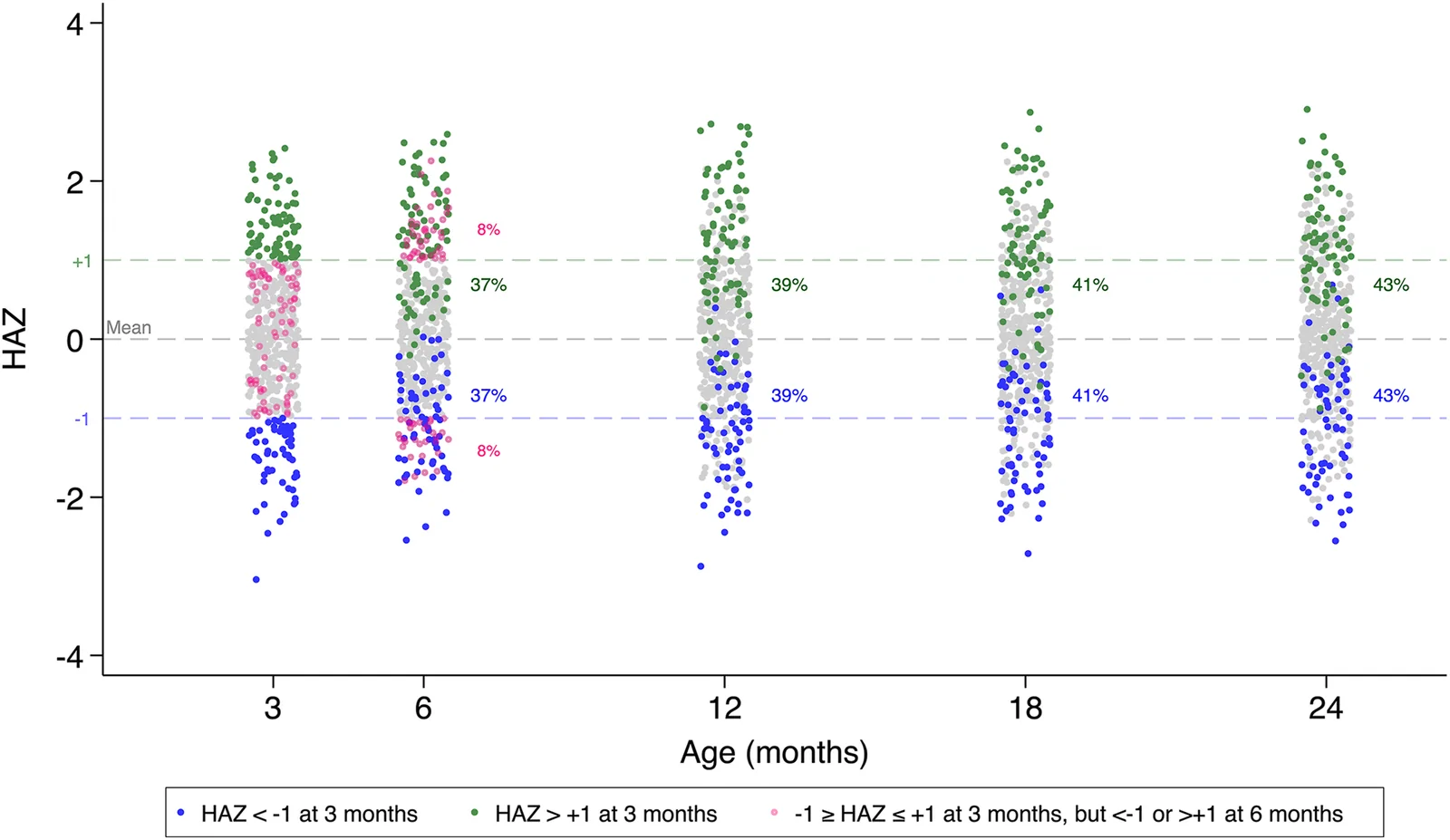
Threshold-crossing events across HAZ thresholds of +1 and −1 in a simulated longitudinal dataset of a population of children with stable mean HAZ = 0 and constant SD of 1.0 from 3 to 24 mo of age (*N* = 100,000). Percentages in green (row of percentages between 0 and +1) denote the number of children who cross down from above to below HAZ = +1 at the age shown, of the total number of children with HAZ > +1 at 3 mo. Percentages in blue (row of percentages between 0 and −1) denote the number of children who cross up from below to above HAZ = −1 at the age shown, of the total number of children with HAZ < −1 at 3 mo. Downward (green) and upward (blue) threshold-crossing events represent RTM. Since variance is constant (SD = 1 at all timepoints), a similar number (but smaller proportion) of children move toward the tails in each interval, so that the total percentage of HAZ values <−1 (or >+1) is ∼16% at every timepoint; for example, children who shifted to the tails at 6 mo (shown in pink at 3 and 6 mo) and the percentages in pink (at 6 mo) denote the number of children who were in the central part of the distribution at 3 mo and cross to either >+1 or <−1 at 6 mo, of the total number of children with −1 ≥ HAZ ≤ +1 at 3 mo. All movements between timepoints including RTM reflect the imperfect between-timepoint correlations of repeated length measurements (Figure 1). A random 0.5% subsample of observations are used in the plot to aid in visualizing threshold-crossing event frequencies at each age. Thresholds of +1 and −1 were selected for illustration purposes to show a higher proportion of crossing events than would occur across thresholds of +2 and −2, given the mean HAZ = 0; however, threshold-crossing event frequencies would be similarly symmetrical for thresholds for +2 and −2. HAZ, height-for-age *z*-score; RTM, regression to the mean.

RTM is caused by natural random fluctuations in the rates of linear growth, and not just technical errors in measurement, but may be labeled as a statistical artifact because it yields prominent patterns of change for which the mechanisms are often misunderstood. To appreciate why the misunderstanding occurs, it is essential to recognize that the variability underlying RTM is present across the entire range of measurements. The imperfect correlation between any 2 timepoints causes changes in the relative rankings of children within the sample across the entire baseline HAZ distribution, not just those who started from the lower tail. In tandem with the seemingly disproportionate catch-up experienced by the shortest children, a mirror-image phenomenon of declining HAZ is happening to some of the tallest children in the upper tail of the HAZ distribution (Figure 2). Moreover, some children from the central section of the distribution necessarily move away from the mean toward the tails in any interval, essentially replacing children who regressed toward the mean (Figure 2). Therefore, when researchers focus narrowly on the unidirectional trends of 1 subgroup of the population (e.g. those in a lower tail who move up across a threshold toward the mean) and ignore RTM as a potential explanation, there is a risk of distorting the significance and causes of these changes. For example, in a study of Bangladeshi children enrolled at ∼18 mo of age, those who were considered stunted (HAZ < −2) or at risk of stunting (HAZ <−1 but ≥−2) were provided dietary supplements for 3 mo; if their follow-up HAZ after the period of supplementation remained below −2 or −1, respectively, they were selected to undergo gastrointestinal endoscopy and mucosal biopsy [29]. The investigators attributed the change in HAZ to the dietary intervention [29], yet among children with baseline HAZ below an arbitrary threshold below the population mean, any upward threshold-crossing change in HAZ could be due to RTM and would have occurred in the absence of dietary management or any other intervention. Similarly, when commenting on their findings in a cohort in Ethiopia, Goddard et al. [30] stated that stunting reversal "may be reflective of the impact of various initiatives in place throughout the country to reduce stunting, which include nutritional interventions (particularly predominant breastfeeding in the first 6 mo of life), improved health care access, sanitation, and education". However, their analysis did not directly assess these factors or consider the extent to which RTM alone accounted for the reversals, which would have been observed in the absence of any of the listed factors. Although it is rewarding to see growth indicators improve and there is an understandable natural inclination to credit medical or public health interventions for these improvements, it is essential to first assess whether RTM may instead explain the observed patterns [31].

**FIGURE 3:**
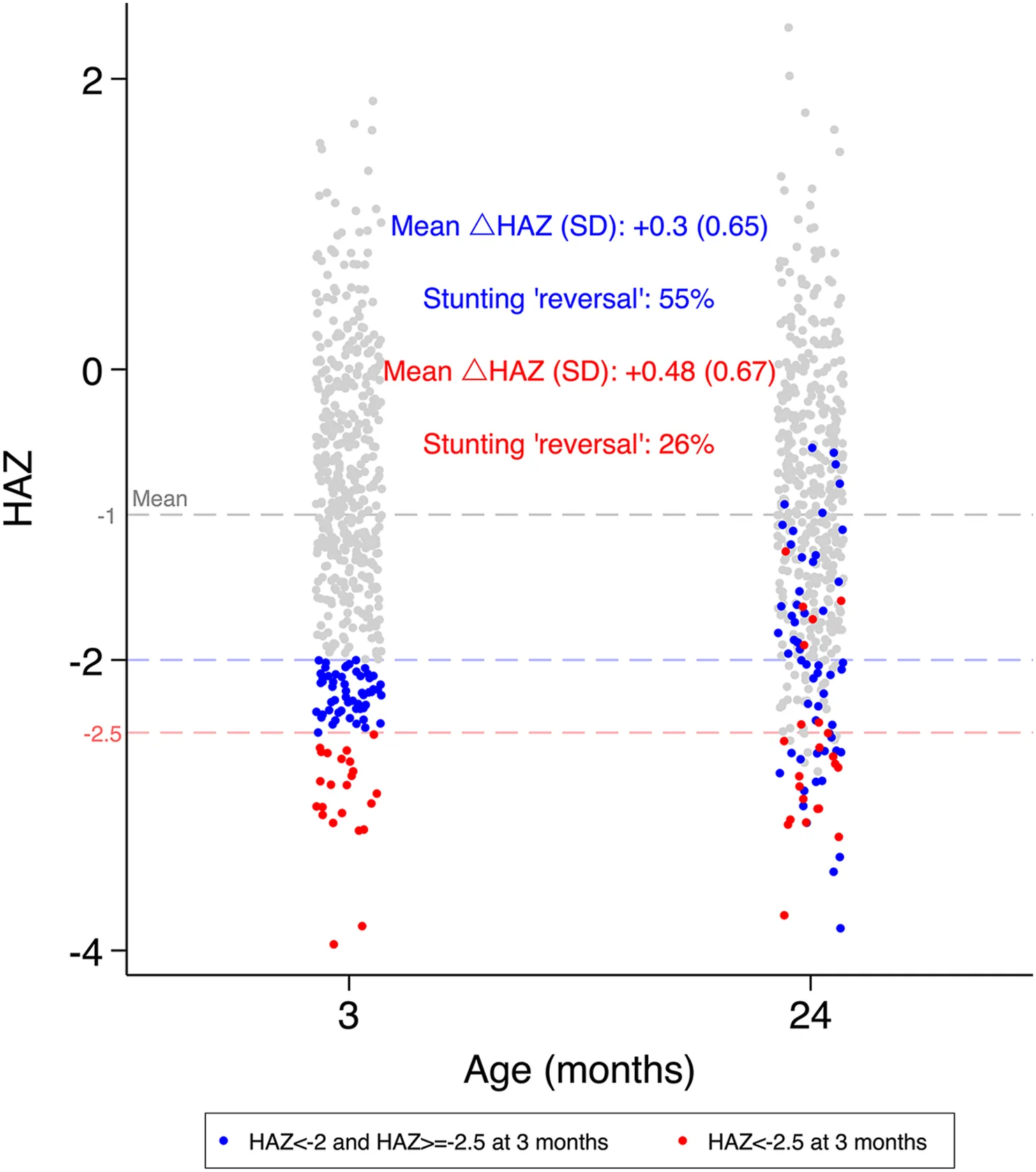
Changes in mean HAZ (ΔHAZ) and frequencies of threshold-crossing events (stunting reversal) from 3 to 24 mo of age in a simulated cohort of children with mean HAZ = −1 (*N* = 100,000). Stunting reversal shown in blue corresponds to the simulated children with HAZ < −2 and HAZ ≥ −2.5 at 3 mo who crossed above the −2 threshold at 24 mo; the proportion is among the total number of children with HAZ < −2 and HAZ ≥ −2.5 at 3 mo. Stunting reversal shown in red corresponds to simulated children with HAZ < −2.5 at 3 mo who crossed above the −2 threshold at 24 mo; the proportion is among the total number of children with a HAZ < −2.5 at 3 mo. All threshold-crossing events reflect regression to the mean due to the imperfect between-timepoint correlation of repeated length measurements (Figure 1). Overall, HAZ at 3 mo was inversely correlated with ΔHAZ from 3 to 24 mo (*r* = −0.35). A random 0.5% subsample of observations are used in the plot to aid in visualizing threshold-crossing event frequencies at each age. HAZ, height-for-age *z*-score.

Threshold crossing is particularly prone to misinterpretation when the thresholds do not have a clinical or biological basis. Among children classified as stunted at a baseline age, the magnitude of the RTM effect affecting each child’s trajectory is influenced by the distance of their baseline value from the population mean as well as the distance to the threshold that defines stunting; consequently, children closest to the threshold are more likely to cross above it (i.e. experience stunting reversal) even though, paradoxically, children farthest from the threshold tend to experience larger magnitudes of between-interval increases in HAZ (Figure 3). Therefore, the particular subset of short children who were stunted and experienced reversal are unlikely to be those with the most impressive improvements in HAZ and, thus, cannot be assumed to be those most responsive to interventions believed to affect child growth; rather, their changes are likely just reflective of the underlying variability in linear growth affecting the entire population.

**FIGURE 4:**
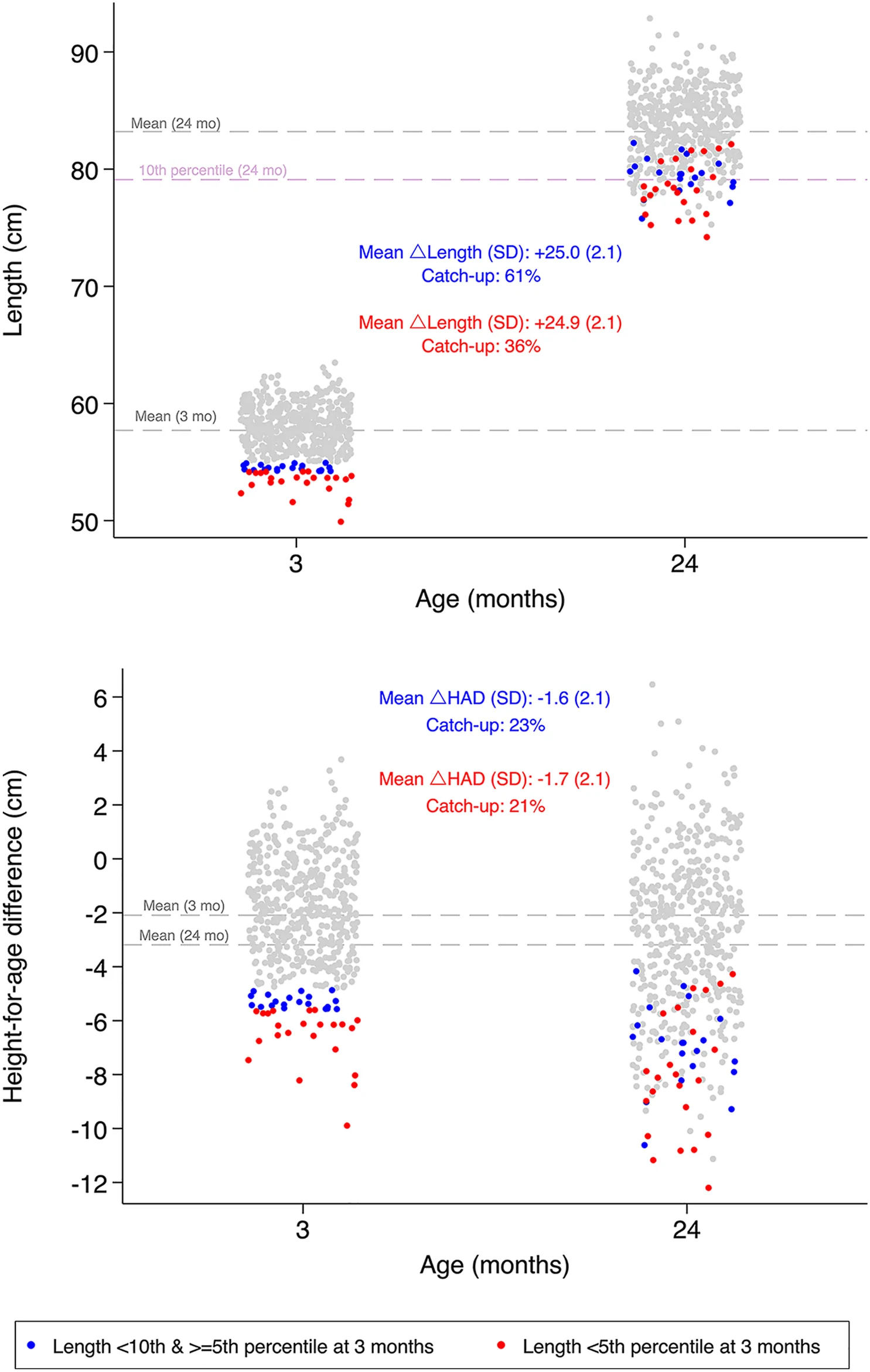
Changes in mean body length (Δlength) or height-for-age difference (ΔHAD) and frequencies of catch-up growth from 3 to 24 mo of age in a simulated cohort of female children (*N* = 100,000). The simulation is analogous to Figure 3 but plotted on the raw length scale (A) or HAD scale (B). Distributions at both 3 and 24 mo have a mean HAZ = −1 and SD based on the WHO Growth Standards (WHO-GS) for girls (length-for-age standards)—at 3 mo, mean = 57.7 cm (SD = 2.1); at 24 mo, mean = 83.2 cm (SD = 3.2). Percentiles are defined empirically for the simulated population (and thus do not correspond to percentiles of the WHO-GS). In (A), the 10th percentile threshold for catch-up was selected arbitrarily to demonstrate changes in subgroups selected from the lower tail of the baseline distribution. Catch-up growth in blue corresponds to simulated children with length less than the 10th percentile and above the fifth percentile at 3 mo who were above the empirical 10th percentile of the population at 24 mo; the proportion is among the total number of children with length less than the 10th percentile and above the fifth percentile at 3 mo. Catch-up growth in red corresponds to simulated children with length less than the 5th percentile at 3 mo who were above the 10th percentile at 24 mo; the proportion is among the total number of children with length below the fifth percentile at 3 mo. In (B), HAD is calculated as the difference between each value shown in (A) and the age-specific median length based on the WHO-GS—3 mo, median = 59.8 cm; 24 mo, median = 86.4 cm. Catch-up growth on the HAD scale is defined as any decrease in the magnitude of HAD (i.e. ΔHAD > 0). All catch-up events reflect regression to the mean due to the imperfect between-timepoint correlation of repeated length measurements (Figure 1). Overall, length (or HAD) at 3 mo was positively correlated with Δlength (or ΔHAD) from 3 to 24 mo (*r* = 0.14); in contrast to the HAZ scale (Figure 3), the expected inverse correlation is not observed on the raw length or HAD scale due to the increase in SD from 3 to 24 mo. Overall mean ΔHAD was −1.1, indicating population-level faltering on the HAD scale despite the stable mean HAZ. A random 0.5% subsample of observations are used in the plots to aid in visualizing threshold-crossing event frequencies at each age. HAZ, height-for-age *z*-score.

RTM is relatively easy to detect in analyses of changes in stunting status, whereby individual children with upward HAZ threshold crossing from below −2 (stunted) to above −2 (nonstunted) are classified as having recovered or experienced stunting reversal (FIGURE 1, FIGURE 3). Yet, due to the mathematical coupling of baseline values with subsequent changes in the same measurement [24], it is important to consider baseline variability and consequent RTM effects whenever one is making inferences about child-level changes in HAZ. Furthermore, RTM may affect any anthropometric measure and can occur on any scale, assuming there is imperfect correlation between each child’s serial measures. Therefore, RTM also affects analyses of raw heights or HAD, not just age- and sex-standardized metrics such as HAZ (Figure 4). The magnitude of observable RTM is scale dependent [32], since the degree to which changes over a defined age interval are correlated with baseline values is affected by the variances of both the baseline and endline measurement distributions; for example, the SD of the raw height distribution naturally increases with age, whereas the HAZ SD remains reasonably constant at ∼1 (by design), such that RTM is usually observed to be of greater magnitude on the HAZ scale than on the raw height scale [23]. Rather than mitigate RTM, the raw height scale partially masks it because of the greater dispersion of raw height measurements as children age (Figure 4). An example of masking is that the correlation between baseline and change (baseline to endline) may be positive on the raw height or HAD scale (Figure 4) whereas on the corresponding HAZ scale, the inverse correlation fulfils the conventional expectation for RTM (Figure 3), although RTM is present on all scales. The challenge in detecting or quantifying RTM when the SD is non-constant is why RTM is typically assessed using standardized measures [32,33]. From a practical standpoint, studies that define threshold heights for which upward crossing is considered to be a form of anthropometric recovery are unlikely to use raw height scales, because any such thresholds are dependent on the expected height for a given age or stage of maturation and, therefore, more readily defined on the sex- and age-standardized HAZ scale. However, a similar phenomenon would be observed on the HAD scale if catch-up growth is defined as a decrease in the absolute magnitude of HAD between 2 measurements such that the follow-up measurement is closer to the median height—per Leroy et al. [11]—among children who were relatively short at baseline (Figure 4B).

## Consideration of RTM in Published Longitudinal Studies of Stunting Recovery, Reversal, or Catch-Up Linear Growth in LMICs

To examine the extent to which RTM has been contemplated in the published literature addressing stunting recovery, reversal, or catch-up linear growth in LMICs, we searched PubMed for observational studies in LMICs published in approximately the last 2 decades (2004 to mid-2024) in which the study findings were interpreted or contextualized in terms of catch-up linear growth (see Supplemental Methods for details of the search strategy, article review and selection process, and the rubric we developed to classify studies with respect to their consideration of RTM effects). Among the 69 articles that described longitudinal studies of height or HAZ (Supplemental Figure 1), we found that a majority (39; 56%) used analytical approaches and/or interpreted their findings in a manner we considered to be at higher risk of RTM artifacts, whereas 30 (43%) used lower-risk approaches in their analyses and interpretations (Table 1). Most of the higher-risk studies (24; 62% of higher-risk articles) used a growth status variable based on a threshold-crossing event (e.g. stunting reversal) or longitudinal modeling with stratification by baseline size (12 studies; 31% of higher-risk articles). Only 14 (20%) articles included narrative statements about RTM; the frequency was similar among higher-risk and lower-risk studies, although most of the studies (4/5) that explicitly used analytical methods intended to address RTM were considered lower risk in our assessment (Table 1). Although the classification rubric had subjective elements and we may have inadvertently misclassified some of the studies (and would welcome correspondence from authors who believe that occurred), the findings suggest that conclusions drawn from many studies of stunting recovery/reversal in LMICs may be affected by RTM artifacts. The findings also indicate that while many authors were evidently aware of RTM and several made efforts to address it in their analyses and interpretations, a substantial proportion of articles on this topic (55/69; 80%) seemed to ignore RTM entirely, even when risk of RTM artifacts may have been high (31/39; 79%). However, an important limitation of the review was that we did not assess the extent to which reported estimates were biased or whether studies would have yielded different conclusions about the determinants and consequences of catch-up patterns had the authors used alternative analytical methods or explicitly considered RTM in the interpretation of their findings.

**TABLE 1:** Inclusion of statements about regression to the mean (RTM) and use of analytical methods that are higher compared with lower risk of RTM artifacts in reports of longitudinal studies of child and adolescent growth in low- and middle-income countries (LMICs), published from 2004 to mid-2024 (*N* = 69 articles)

Parameter	*N*	No mention of RTM	Mentions RTM briefly and not in a manner that qualifies or explains methods or results	RTM is discussed as a factor that may affect or explain methods, results, and/or conclusions and interpretation of the results	Analysis is performed in a manner explicitly intended to address RTM (e.g. sensitivity analysis or RTM correction)
*N*	69	55	2	7	5
Higher risk of RTM artifacts^1^, *n* (%)	39	31 (79)	2 (5)	5 (13)	1 (3)
Change in growth status based on a *z*-score threshold-crossing event^2^	24	19	2	3	0
Longitudinal models with stratification by baseline *z*-score^3^	12	11	0	1	0
Lower risk of RTM artifacts^1^, *n* (%)	30	24 (80)	0 (0)	2 (7)	4 (13)
Conditional growth modeling^4^	6	5	0	0	1

1 Studies considered higher risk of RTM artifacts used ≥1 of the following types of analyses: growth variable was defined by a *z*-score threshold-crossing event (e.g. stunting reversal or recovery), without correction for RTM; analyses or graphical representations of children grouped by (or selected on) baseline size; growth was the outcome variable and baseline *z*-score was an exposure variable of interest; growth was the exposure variable in a regression model without adjustment for baseline size; and longitudinal modeling of serial size measures (e.g. mixed model) with stratification by baseline size or interactions between age and baseline size that yielded inferences about associations of growth rates with baseline size. All other methods were considered lower risk of RTM artifacts. See Supplemental Methods for details of the rubric and the classifications of individual studies.

2 Coded as 4b-2 in the rubric (Supplemental Methods). Nearly all studies used the terms stunting reversal and/or recovery from stunting; infrequently, synonyms such as improvement or transition (from stunted to nonstunted status) were used to describe the *z*-score threshold crossing. None of these studies used methods to correct for RTM.

3 Coded as 5-5 or 5-6 in the rubric (Supplemental Methods).

4 Coded as 3-4 in the rubric (Supplemental Methods).

## Statistical Methods to Address RTM in Analyses of Child Linear Growth in Longitudinal Cohorts

In randomized controlled trials (RCTs), RTM is readily addressed when endline HAZ (or change in HAZ) is compared across randomly assigned intervention groups, because baseline HAZ is uncorrelated with the intervention assignment. In RCTs in which baseline HAZ differs across groups despite randomization, adjusting for baseline (pretreatment) values (often referred to analysis of covariance, ANCOVA) is usually a valid approach that yields unbiased estimates of the causal effect of a well-defined exposure (the experimental intervention) on HAZ [34].

Conversely, it is less obvious as to how to best handle RTM in observational studies, which are more susceptible to the effects of RTM than RCTs. In principle, RTM effects can be managed when analyses include all children in the population rather than focus on ≥1 subgroups selected on or defined by baseline HAZ and when baseline HAZ is not itself considered as a determinant of subsequent changes in HAZ. However, there is a wide range of child growth modeling methods [35,36], and determining the extent to which each method can mitigate RTM is challenging, particularly as it is related to the interpretation of coefficients, not just model specification (see Supplemental Methods for details of our approach to the classification of methods with respect to their risks of RTM artifacts). Model choice also depends on many competing considerations, not just concern about RTM; for example, adjustment for baseline HAZ as a covariate in regression models (similar to ANCOVA models used in RCT analysis) may mitigate RTM effects [37], yet this advantage can be outweighed by an overadjustment bias if baseline HAZ is a mediator of the effect of risk factor of interest on later HAZ [38,39].

Conditional growth modeling is a relatively common approach in longitudinal observational studies of child growth (i.e. when the individual child measurement is the unit of analysis), particularly when HAZ in early childhood is considered as a risk factor for health outcomes later in life [35,36]. It is widely considered to be a suitable method to manage RTM; however, its uses in quantifying catch-up merits some cautionary notes, and a brief examination of conditional models highlights challenges that researchers face in attempting to address RTM when studying catch-up patterns in observational studies. The following discussion of conditional models as applied to longitudinal cohort studies also serves to set the stage for a later discussion of such models in the context of population-level catch-up analyses.

A general form of an equation to estimate conditional HAZ at 1 timepoint (t_2_) given measured HAZ at an earlier timepoint (t_1_) is based on the regression of the endline HAZ (Z_2_) on baseline HAZ (Z_1_), as follows [40]:

(1) $$Z_{2}=a+b\left(Z_{1}\right)+e$$

In Equation *1*, *a* is the intercept (i.e. average value of Z_2_ when Z_1_ = 0), and *b* is the slope of the average change in Z_2_ for a 1-unit change in Z_1_, assuming a linear relationship between Z_2_ and Z_1_. The individual child-level residual (e) represents the deviation of an individual child’s Z_2_ from the expected value given their observed Z_1_ and, therefore, captures the child-level variation at t_2_ not explained by Z_1_. The residuals (e) can be interpreted as conditional HAZs and used in downstream analyses as RTM-corrected relative measures of the size of each child at t_2_, given their size at t_1_. It is important to note that this model assumes that RTM is movement toward the empirical cohort mean HAZ rather than an externally defined benchmark such as the WHO-GS median. If group-averaged predictions are of interest, Equation *1* can be expressed in terms of the expected (E) values of Z_2_, given Z_1_ for the observed population, as follows:

(2) $$E\left(Z_{2}\right)=a+b\left(Z_{1}\right)$$

As described by Cole [40], an even simpler form of Equation *2* can be derived in the special case if the expected HAZ at endline equals zero when the baseline HAZ is zero [i.e. E(Z_2_) = 0 when Z_1_ = 0], such that the model intercept is also zero (*a* = 0) and the sample standard deviations (s) of both Z_1_ and Z_2_ are equal, so *b* equals the correlation coefficient (*r*) between Z_1_ and Z_2_ [40], as follows:

(3) $$E\left(Z_{2}\right)=r\left(Z_{1}\right)$$

This set of assumptions that give rise to Equation *3* are fairly stringent but may be reasonable for a healthy population with a HAZ distribution that has a consistent mean HAZ approximately equal to 0 and is normally distributed with a consistent *s* approximately equal to 1. However, these special case conditions do not apply to most LMIC populations, particularly since mean HAZ is usually less than zero and declines with age. This is a key consideration that may be easily overlooked; for example, in 1 of the studies identified in the literature review, the reduced form of Equation *3* was applied to a study of several cohorts in LMICs [41]. The application of Equation *3* was further complicated by the authors’ use of an *r* value calculated for "the subset of children who were stunted at baseline and not at endline" [41], even though *r* (or *b*) should be estimated using the entire observed population (i.e. including both stunted and nonstunted children) [21]. Only the general form of the conditional growth model (Equation *1*) is suitable to address RTM in longitudinal studies of child growth in LMICs when inferences are based on between-child comparisons of growth trajectories within a defined cohort. Conditional growth models inherently treat variation in baseline HAZ as a nuisance rather than a characteristic of interest, so the approach cannot be used in an attempt to mitigate the effects of RTM in analyses in which children have been subgrouped or selected according to baseline HAZ values or HAZ threshold-crossing events. For researchers who question the validity of the HAZ scale for linear growth trajectory modeling, conditional growth analyses can be performed using other measurement scales (e.g. HAD; age-standardized *z*-scores generated from a local growth reference; and *z*-scores calculated using the cohort mean and SD).

Despite the stated concerns with subgrouping children based on HAZ thresholds, we acknowledge that some researchers may still be inclined to describe the growth of a subgroup of children selected because they were below a defined HAZ threshold in the baseline measurement distribution. A potential approach in this scenario could be to quantify the RTM effect, defined as the average change in a measurement in the subgroup from baseline to a follow-up measurement timepoint due to RTM [21]. The expected change due only to RTM can be compared with the subgroup’s observed change, to estimate the extent to which the observed change was attributable to RTM rather than risk factors or other biological phenomena of interest. See Linden [21] and Thomas et al. [22] for detailed discussions of the conventional formulas used to calculate the RTM effect. However, in the context of whole-population linear growth faltering, age-related changes in HAZ values are also influenced by the overall downward shift in the population HAZ distribution. The conventional published formulas for estimating the RTM effect do not account for this shift [21,22] and can therefore lead to erroneous estimates of the changes in HAZ in the stunted subgroup that would be expected to result from RTM (Figure 1B).

Another approach closely related to the calculation of the RTM effect would be to undertake simulations like those we have used to generate the figures in this article (Supplemental Methods). Simulations enable the estimation of frequencies of stunting reversal attributable to RTM, given the statistical properties of baseline and endline HAZ distributions and the correlation between paired baseline and endline values; those estimates can then be compared with the corresponding observed proportion of children who empirically reversed in each defined interval. For example, we demonstrated that, in a large multicohort study of early childhood linear growth in LMICs, the phenomenon described by the authors as stunting reversal [18] was probably entirely an artifact of RTM because simulated frequencies of reversal due only to RTM were similar to or higher than the corresponding observed proportions [19].

Estimating the RTM effect or conducting simulations as described earlier would be improvements over the reporting of reversal/recovery frequencies in a manner that does not acknowledge or account for RTM (Table 1), yet we still recommend against any use of HAZ threshold-crossing measures (stunting reversal or recovery) to characterize catch-up growth. The basis for this recommendation was explained in the preceding section, and the core reasons can be summarized as follows: *1*) downward whole-population shifts in the HAZ distribution in early childhood in most LMICs imply nearly all infants, and children are growing more slowly than expected for their chronological age [3]; therefore, neither stunting nor reversal of stunting are meaningful classifiers at the individual level; *2*) observed stunting reversals are likely to be attributable to RTM because in most populations, and the threshold (HAZ = −2) is well below the population mean in the lower tail of the HAZ distribution; *3*) stunted children closest to the threshold are most likely to cross above it even though other children with even lower baseline HAZ may experience larger increases in HAZ in the same interval (Figure 3); and *4*) instances of true catch-up growth based on clinical criteria would be expected across the full baseline height distribution, not only among children classified as stunted at baseline; therefore, even if analyses based on upward threshold-crossing among stunted children are corrected for RTM, these would still entirely ignore recovery or catch-up growth among nonstunted children [15].

## RTM in Studies of Population-Level Changes in Average HAZ

Thus far, we have primarily considered RTM in the context of epidemiological cohort studies in which the unit of analysis is the individual child measurement, repeated for each child at multiple age timepoints. That discussion focused on RTM as it pertains to age-related changes in HAZ of individual children, including when individual children are classified based on whether their change in HAZ involved the crossing of a threshold (e.g. reversal). However, it is also important to consider RTM when the unit of analysis is a whole population at 1 time and the analyses focus on population-level changes in mean HAZ (or other population-average measures such as mean HAD or growth delay). The availability of population-representative survey datasets from most LMICs, such as Demographic and Health Surveys (DHS), has enabled numerous studies of growth faltering based on analyses of age-binned data or pseudolongitudinal approaches that quantify trends by age or calendar date (i.e. secular changes) [1,42]. When analyzing either age-related or secular trends, population-level catch-up growth is often conventionally considered to occur when mean HAZ or mean HAD increases from a negative value toward zero or where growth delay plateaus; however, these definitions often lead to differing conclusions about whether catch-up has occurred [8].

In a methodological study of population-level catch-up growth and RTM, Cameron et al. [23] proposed that in settings where the population mean height or HAZ is below the median of the international growth reference/standard, changes in population mean height/HAZ with age will be subject to RTM, even in the absence of true catch-up. To account for RTM in this context, Cameron et al. [23] adapted coauthor Cole’s [40] conditional growth model, which is more commonly used in analyses of child-level data as described earlier (Equations *1*–*3*) and applied the simplified form of Cole’s original equation to define population catch-up on the HAZ scale, whereby:

(4) Population catch-up growth = meanZ_2_ – *r*(meanZ_1_)

In Equation *4*, *r*(meanZ_1_) reflects the endline mean HAZ that would be expected only because of RTM and meanZ_2_ is the observed endline mean HAZ; thus, the difference between these terms represents the true catch-up (toward mean HAZ = 0) not explained by RTM [23].

There are several assumptions implicit in this approach that raise questions about its suitability for quantifying population-level catch-up in LMICs. First, Cameron et al. [23] assumed that the LMIC population is selected from the tail of the international reference distribution of heights of individual children, such that RTM causes a population mean HAZ to tend to move toward the international median height (HAZ = 0). However, a population mean HAZ should be considered to arise from an underlying distribution of populations, the mean and variance of which are unlikely to be analogous to those of a child growth reference or standard distribution such as the WHO-GS. Since mean HAZ is less than zero across nearly all LMICs, the magnitude and direction of any population-level RTM effect may be predominantly toward an empirical (and nonzero) grand mean of a distribution of mean HAZs of local or regional population(s) from which the survey sample was drawn, rather than the international normative median based on a child growth standard. A second assumption of Equation *4* as implemented by Cameron et al. [23] was that child-level correlation coefficients (i.e. values of *r* estimated within a cohort) could be used in analyses of population-level trajectories; this assumes that the correlation between individual child HAZ values measured at 2 ages is the same as the correlation between pairs of population mean HAZ estimates for the same interval. It seems unlikely that such a direct correspondence exists, and although there are published individual child-level correlation matrices [26], we are unaware of a population-level correlation matrix that could be used to test this assumption. A third assumption of Cameron et al. [23] was that population trajectories by age can be measured on a HAZ scale based on a child growth reference or standard. As mentioned earlier, we previously demonstrated the limitations of using mean HAZ (or HAD) trajectories to quantify age-related patterns of population-average growth. Conversely, mean HAZ appears to be suitable for tracking secular trends (assuming a consistent population age distribution) [8]. Therefore, we considered an alternative approach to that proposed by Cameron et al. [23], whereby we adapt the general form of the conditional modeling approach (Equation *1*) to estimate population-level secular trends (i.e. changes in a population along a calendar time scale) as follows:

(5) $${\text{meanZ}}_{2}=a_{p}+b_{p}\left({\text{meanZ}}_{1}\right)+e_{p}$$

In Equation *5*, the variables *a*_*p*_, *b*_*p*_, and e_*p*_ are interpreted as for Equation *1*, but at the population level, whereby *e*_*p*_ is a population-level residual, representing the deviation of a population’s mean HAZ at time 2 (meanZ_2_) from the expected value given its observed mean HAZ at time 1 (meanZ_1_), such that e_*p*_ > 0 could be interpreted as catch-up relative to other populations, and e_*p*_ < 0 would represent relative faltering. To implement this method, one would need to use a multisurvey dataset to estimate *a*_*p*_ and *b*_*p*_, whereby the unit of analysis is the population and the data are drawn from multiple populations/countries with ≥2 surveys in different years from each population/country. A similar approach could be used to model between-country variations in secular changes in raw heights, HAD, height-age, or growth delay, but future research is required to examine and validate such approaches.

Twenty years following the article by Cameron et al. [23], there is a lack of consensus about the importance of RTM in analyses of population-level linear growth trajectories or whether it even needs to be addressed. For example, in explaining their analyses of mean HAD- and HAZ-by-age trajectories in DHS and selected cohort datasets, Leroy et al. [20] wrote, “Given our focus on population-level catch-up growth in height, RTM does not affect our analyses” and cited Cameron et al. [23], suggesting that Leroy et al. [20] disagreed with the core premise of Cameron et al. that RTM affects population-level trajectories [23]. More recently, Leroy et al. [11] reported a meta-analysis of observational studies of group-average catch-up of children from LMICs who were adopted or placed in foster care in a higher-income setting, whereby catch-up was primarily quantified on the HAD scale but also reported on the HAZ scale [11]. Although the authors did not acknowledge RTM effects or refer to Cameron et al. [23], the age-related changes in mean HAD in their meta-analysis were precisely the types of population-level estimates for which Cameron et al. [23] had advocated for consideration of RTM. The effects of RTM in such analyses are difficult to quantify, but its absence would require that baseline mean HAD is perfectly correlated with follow-up mean HAD, which seems implausible; therefore, to some unknown extent, RTM would have influenced estimates of overall group-level catch-up by Leroy et al. (mean change in HAD), not just the age- or sex-stratified analyses in which the subgroups differed by baseline HAD (i.e. RTM effects on within-cohort comparisons, as described earlier). Nonetheless, there is no established method to quantify or correct for population-level RTM effects in this analytical context; even if one simply assumes that the RTM effect is toward the international median based on the WHO-GS (i.e. HAD = 0), the relevant population-level correlation coefficients are unknown and unlikely to be reliably estimated from the dataset used for the meta-analysis itself (i.e. 9 relatively small cohorts of institutionalized children) [11]. To bolster the findings based on the observational studies, Leroy et al. incorporated the analysis of a randomized trial, which is a design that guards against RTM as discussed earlier. Another approach would be to conduct sensitivity analyses in which varying degrees of RTM correction are introduced to establish the correlation thresholds at which the observed catch-up would exceed that predicted only by RTM.

## Conclusions

In longitudinal cohort studies of child growth in LMICs, RTM always arises, for all anthropometric measures and on all scales. In analyses of catch-up growth, RTM is especially pronounced in analyses of subgroups of children selected from the tail of the HAZ distribution, such as those classified as stunted. It is particularly apparent when the trajectories of such children are expressed as HAZ threshold-crossing events such as stunting reversal or recovery. Missing or misinterpreting RTM can lead researchers to overestimate catch-up patterns and incorrectly attribute them to dietary, medical, or public health interventions. Statistical methods such as conditional growth modeling can be used to correct for RTM in some situations, but there is no method that overcomes all of the conceptual and statistical pitfalls of analyses based on HAZ threshold-crossing events or other types of catch-up among children selected from a lower tail of the height distribution, irrespective of the measurement scale.

In studies of population-level growth trajectories (e.g. tracking mean HAZ or mean HAD), RTM also occurs but is often more difficult to detect or address compared with child-level analyses of longitudinal cohorts. In the context of multisurvey/country studies, RTM can potentially be mitigated using statistical methods such as conditional growth modeling. However, there is no standard approach to addressing RTM when considering the catch-up patterns of a single population or small number of cohorts, and while more research is needed to guide researchers in this context, it would be reasonable in the meantime for researchers to acknowledge how observed trajectories may be subject to RTM and interpret them accordingly.

Peer review has an important role in detecting RTM that has been otherwise overlooked. We encourage journal editors to routinely screen submissions for RTM artifacts and provide guidance to submitting authors regarding analytical approaches that could be used in a revised submission. Our literature review revealed that many published studies of stunting reversal or recovery over the past 2 decades have ignored RTM, thereby putting into question the existing evidence regarding linear catch-up growth in LMICs. Ideally, authors of published studies will revisit their previous studies of stunting recovery and reversal and, where possible, remediate the scientific record. In future research, we advise against the use of threshold-crossing event analyses or outcome measures such as stunting reversal and recovery. In analyses of catch-up growth, researchers should strive to use lower-risk methods that quantitatively account for RTM effects or that enable an assessment of the extent to which the observed patterns may be attributable to RTM.

## Author contributions

The authors’ responsibilities were as follows—DER, DGB, and KMW designed the research and wrote the paper; DER, DGB, KMW, and HQ conducted the research; DER and DGB: had primary responsibility for final content; and all authors: have read and approved the final manuscript.

## Declaration of Generative AI and AI-assisted technologies in the writing process

The authors declare that no generative AI or AI-assisted technologies were used in the writing of this manuscript.

## Funding

This work was supported in part by the Canadian Institutes of Health Research (CIHR) (project grant 169133). The CIHR had no involvement in this study and does not restrict works for publication.

## Data availability

Analytic code scripts used in the simulations described in the manuscript are publicly available at https://github.com/DGBassaniLab/LGF_RTM.

## Conflict of interest

The authors report no conflicts of interest.
