# Supplementary material for: Stunting Reversal versus Regression to the Mean in Epidemiological Studies of Child Linear Growth in Low- and Middle-Income Countries
Source: Adv Nutr. 2026 Jun 20;17(8):100688. doi: 10.1016/j.advnut.2026.100688 (PMC13494613; doi:10.1016/j.advnut.2026.100688)
Supplement: Supplementary file 1 [file mmc1.pdf]

Stunting reversal versus regression to the mean in epidemiological studies of child linear growth in low- and middle-income countries

Daniel E. Roth et al.

SUPPLEMENTARY MATERIAL

Table of Contents:

Supplementary Methods: Scoping review 2

Supplementary Figure 1. Study selection flowchart 10

Included studies 11

Supplementary Methods: Simulations 14

References cited 16

Scoping review methods

I. Literature Search

The aim of the scoping review was to assess how often and using what methods RTM is handled in data analyses and discussed by authors in their reports of observational epidemiological studies of catch-up growth of children and/or adolescents in low- or middle-income countries (LMICs) that were published in the past two decades. A search was conducted in PubMed on May 27, 2024 using the following search terms:

> (growth disorders[MeSH Terms] OR stunting or "linear growth faltering") AND (reversal OR recovery OR catch-up OR "regression to the mean" OR "regression toward the mean" OR Recurrence[MeSH Terms]) AND (child* OR children OR pediatric) AND (developing countries[MeSH Terms] OR "LMIC" OR "low- and middle-income" OR Africa[MeSH Terms] OR Asia[MeSH Terms] OR Latin America[MeSH Terms])
> **Filters**: 2004-2024; Humans

As a scoping review of a methodological issue, the goal was to broadly consider a wide range of studies of catch-up growth in LMICs but not necessarily identify every study published on this topic. The inclusion of the search terms "regression to the mean" and "regression toward the mean" was intended to identify relevant studies that explicitly addressed RTM, even if they were not identified by any of the other growth-related keywords; in theory this would bias the strategy towards rather than away from studies that addressed or acknowledged RTM.

We considered articles to be eligible for inclusion if they met the following inclusion criteria:

1) Study population included infants, children, or adolescents living in or recently emigrated from a country classified as a low-, lower-middle, or upper-middle income country according to the World Bank income classifications at the time of the study (https://datahelpdesk.worldbank.org/knowledgebase/articles/906519-world-bank-country-and-lending-groups); international adoption studies were considered eligible even if the first measurement used in the analysis was obtained upon or soon after arrival in the destination country;
2) Longitudinal observational study (or a meta-analysis of such studies) of height or height-derived measurements using data obtained at 2 or more age time points for each participant (data could be drawn from a randomized trial if analyses were not based on the randomized design);
3) Described or analysed an age-related change or trajectory of a length- or height-related growth parameter such as height, height-for-age z-score (HAZ), height-for-age difference (HAD), or used a classification of each child based on a linear growth status (e.g., stunting) or pattern (e.g., recovery from stunting);
4) Presented the above-mentioned measure(s) of linear growth using graphs or descriptive statistics, and/or used the measure(s) as an exposure or outcome variable in an analysis of epidemiological associations;
5) Used or interpreted the above-mentioned growth variable and/or results from its analysis as the basis for inferences or comments regarding linear growth catch-up, stunting reversal, recovery from stunting, improvement, or using other terms that similarly signified a catch-up pattern of linear growth.

We excluded case reports of individual children or case series of patients evaluated or treated for specific medical conditions (e.g., asthma, diabetes) although we did not exclude studies of cohorts partly or entirely comprised of children with the following conditions: HIV exposure or infection; moderate acute malnutrition (MAM) or severe acute malnutrition (SAM); fetal growth restriction or low birth weight (LBW); or preterm birth. We also excluded reports of randomized controlled trials in which the analysis of catch-up growth was based only on comparisons between treatment groups to which participants had been randomly allocated.

The search yielded 247 articles, with no duplicates, of which 126 were selected for full-text review, and 69 were included in the detailed assessment of RTM handling and RTM-related comments (See accompanying supplemental table). We initially considered including cross-sectional studies or surveys that involved pseudo-longitudinal analyses of secular or age-related trends in a height or height-derived measure, but otherwise met the inclusion criteria; however, among the 126 articles initially selected for full-text review, only 2 such studies were identified(1, 2), and they were ultimately excluded from the focused review of RTM handling and comments because we were unable to apply the same classification/rating scheme to such studies as was used for the longitudinal studies. We initially considered commentaries or narrative review papers focused on linear growth in our full text review but only included articles with quantitative longitudinal analyses of anthropometric data (per inclusion criteria above) in the analysis of RTM handling and comments. Lastly, we identified two studies that generally met the inclusion criteria but were methodological studies focused on RTM(3, 4); as these studies could not readily be classified in a similar manner as other longitudinal studies, they were excluded from the final analytical set.

Screening, full-text review and data abstraction was conducted using Covidence(5). Each article was independently screened, reviewed and abstracted by at least two authors. Discrepancies were reconciled by a third author and by consensus attained at co-author team meetings. The following information was extracted: Study design, data source(s) (e.g., name of cohort), sample size, country, ages at which data were collected, linear growth-related measurements and variables, terms and definitions to describe linear growth patterns (e.g., catch-up, reversal, recovery), statistical method(s) used to analyze age-related linear growth, and comments related to RTM in the article text including table footnotes.

II. Study Assessment and Classification

We aimed to classify each study in terms of how RTM was managed in the analysis, as well as the extent to which the authors acknowledged RTM as a methodological issue that could affect their results and conclusions. Initial reviews revealed that in many studies that employed methods that were lower risk for generating RTM artifacts, the authors did not discuss RTM as a methodological consideration (i.e., reason for selecting a particular method). Therefore, we recognized the two issues (RTM handling in the analysis and discussing RTM in the text) should be considered separately. We undertook an iterative process to develop a classification approach for both elements, as outlined below. Classification of each article was initially performed independently by at least two authors; however, as we conducted multiple rounds of reviews and a relatively small proportion of the articles received most of the attention in team discussions, it was unfeasible to remain completely independent as we worked towards consensus regarding the classifications.

Part A: Whether and how RTM is mentioned in the paper

This part refers to whether and how the term 'regression to the mean' or 'regression toward(s) the mean' or 'mathematical coupling' was used in the paper text (including table footnotes or figure legends). The classification is based on explicit mentions of RTM, acknowledging the possibility that authors may consider RTM without mentioning it directly (e.g. "we did this to account for the artifactual correlation between baseline and subsequent change").

Studies should be classified as:

1) **No mention of RTM**.
2) **Mentions RTM briefly** and not in a manner that qualifies or explains methods or results.
3) **RTM is discussed as a factor** that may affect or explain methods and/or results and/or conclusions and interpretation of the results.
4) **Analysis** is performed in a manner specifically and explicitly **intended to address RTM** (e.g., sensitivity analysis, RTM correction).
5) **Methodological study focused on RTM effects** [Note that papers are not further considered in Part B].

Each study should be classified at level A as 1,2,3,4 or 5. The classification is hierarchical; higher levels (such as 4) supersede lower levels (e.g. if a study meets criteria for both category 3 and 4 criteria, the final classification will be 4).

Part B: RTM risk and management within the statistical analysis related to catch-up growth, recovery or reversal or stunting

The focus of this part is on the statistical analysis or graph that is used to make inferences about catch-up growth, recovery or reversal or stunting, irrespective of the extent to which the authors did or did not acknowledge or discuss RTM (per Part A). Here, the extent to which authors addressed RTM is based on what is feasible within the context of their available data, so refers to inferences about patterns of growth in subgroups or variations in growth within the studied population. Although RTM effects at the population level (i.e., comparing *across* populations) are relevant to the broader discussion of RTM artifacts, these would not typically be expected to be manageable in the analytical framework of a study based on individual child-level analyses. If population-level RTM artifacts are discussed by authors (e.g., in the Discussion section of the paper), then this should be captured in Part A (e.g., response 3, 'RTM is discussed as a factor that may affect or explain methods and/or results).

The assessment of the extent to which RTM artifacts are managed in the analysis is approached in a stepwise manner. Where there is mention of a height-for-age z-score (HAZ) in the guidance below, this could be substituted for another measure of stature (e.g., raw height, length, height-for-age difference).

Methods considered higher-risk for RTM artifacts are indicated with a double asterisk (**).

***Step 1*: Identify the analysis(es) relevant to the discussion of catch-up growth.**

There is no response required at this step, but this is a prompt for the reviewer to ensure that a relevant analysis (or analyses) can be clearly identified, and which will be the basis for classification. If studies report multiple analyses, those that are primarily relevant to the catch-up growth-related inferences are the subject of classification. If multiple analyses are relevant, and cannot be otherwise prioritized, then the higher-risk analysis will be used for classification purposes.

***Step 2*: Does the study involve the derivation of a new child-level growth measure or variable using serial anthropometric data (at least 2 measures per child) and use this new variable in the analysis?**

This step determines if the study involved creation of a new variable, calculated for each child, based on serial anthropometric data (i.e., 2 or more measures per child). New variables would represent a child-level measure of growth (i.e. change in size with age) and each child has a value for this new variable, for a given age interval.
Examples include individual change or individual slope or individual classification as recovering, catching-up, crossing a threshold in the HAZ scale or gaining/losing a certain pre-specified quantity on the HAZ or height scale.

> ONLY if the answer to Step 2 is **Yes**, proceed to **Step 3.**
> If the answer to Step 2 is **No**, proceed to **Step 5** and classify the study based on the description provided for each category**.**

***Step 3*: Classify the derived child-level growth variable**

If a child-level growth variable is derived, classify the variable as follows and proceed as indicated for each type:

> **Type 1: Change in size** (i.e., delta, change score) **or individual level slope**.
> Proceed to Step 4a.
>
> **Type 2: Categorization based on attaining a minimum magnitude of change in HAZ or the individual level slope, or a magnitude of change/slope in a defined range of values,** in a given age interval. This is a categorical variable derived from the type of variable mentioned in Type 1, indicating whether the child met some defined criteria for catch-up/reversal/recovery (e.g., change in HAZ equals a certain pre-specified value). A critical feature that distinguishes this from Type 3 is that Type 2 can be defined for all children **irrespective of their baseline z-score**.
> Proceed to Step 4a.
>
> **Type 3: Change in status based on a z-score threshold-crossing event**. This is a categorical variable that indicates whether the child met some defined criteria for catch-up/reversal/recovery. The critical feature is that this can only be defined for children below a defined z-score cut-off at baseline, indicating a sub-group was selected using HAZ in a preceding observation time (usually baseline, but not restricted to baseline). This approach inherently ignores RTM but could be addressed in a subsequent step using an RTM

correction method or using a simulation to understand whether the observed effect is more or less than expected based on RTM.
Proceed to *Step 4b*.

Type 4: Conditional growth measure, which is a relative size at one time point, accounting for size at a prior time point. This is typically based on a regression of size (e.g., HAZ) at a second (or subsequent) timepoint (t_{n+1}) on size (HAZ) at the baseline or first (or preceding) timepoint (t_n), and using the model residuals as the (RTM-corrected) relative measures of each child's size at t_{n+1}. Alternatively, this could be done using the conditional random slope approach (Leung et al 2017). If this derived conditional measure is used in the analysis (as an outcome or exposure), this approach would fully mitigate RTM artifacts. So, if the study is classified as type 4 at this step, no further classification us required (*i.e. classification* Stops here).

Step 4a: How is the growth variable modelled or visualized? [Only for studies classified as Type 1 or Type 2 in Step 3]

If there is an initial derivation of an individual-level growth variable but the variable is a continuous slope/change (Type 1 per Step 2) or classification based on a magnitude of change/slope (Type 2 per Step 2), then further classify the modelling or visualization approach relevant to catch-up inferences as follows:

1) **Growth variable is an outcome, in a model with adjustment for baseline z-score.**
Use of the growth variable as an *outcome* in a model that adjusts for baseline z-score (i.e., ANCOVA model) would be relevant if the study focuses on risk factors associated with the growth variable. In such models, the baseline z-score is *not* a factor of interest but rather a nuisance. If the study reports the association between baseline values and change in height (HAZ), RTM effects are exacerbated, and the classification of the study should be #5 (below).

2) **Population-average summary of the growth variable.**
Expression of a population-average summary (e.g., mean or median) of the individual-level growth variable, or depiction of this variable graphically for the group as a whole.

3) **Growth variable as an exposure, in a model with adjustment for baseline z-score.**
Where the growth variable is an *exposure* variable in a model that adjusts for baseline z-score, the dependent variable is some other non-height-related outcome, and the investigators are interested in whether catch-up growth is related to (as a risk or determinant of) that outcome. Here, baseline z-score is a nuisance/confounder, and any inferences regarding the growth variable (i.e., delta/change) are adjusted for baseline.

4) **Growth variable is an outcome, but there is no adjustment for baseline z-score in the model.**
Where the growth variable is an outcome in a model in which baseline z-score is **not** included as a covariate, the model is similar to approach #1 (above), but does not account for variation in the growth outcome due to baseline z-score. Inferences about other risk factors may be confounded by baseline HAZ, which would be subject RTM effects. However, there may be a justification for not including baseline as a covariate if

there is concern about 'over-adjustment bias', particularly if baseline is a mediator of the effect of the exposure of interest as discussed by Kramer et al 2016(6).

5) ****Growth variable in the outcome, and baseline z-score is included as covariate of interest.**
This model is structurally similar to #1 (per above), but baseline z-score is considered a determinant or risk factor of interest, rather than a nuisance or a variable used for adjustment of other estimates. Inferences about the effect of baseline z-score (or the effects of the classification of children using baseline height measures) on a subsequent change in z-score are subject to the inherent mathematical coupling of change with the baseline value, and therefore the approach is considered at higher-risk for RTM effects.

6) ****Growth variable is the exposure, in a model not adjusted for baseline z-score.**
This is structurally similar to model #3 but potential confounding by baseline z-score is not considered, so inferences about growth could be due to the coupling of baseline with subsequent growth. Therefore, this approach is considered to be at higher risk for RTM artifacts.

7) ****Population-average summary by subgroups defined by baseline z-score.**
Expression of a population-average summary (e.g., mean or median) of the individual-level growth variable by subgroups defined by baseline z-score or some other variable that is associated with baseline z-score (or depiction of the same in graphical format). Given the mathematical coupling of baseline with subsequent change, the subgrouping by baseline z-score makes this approach higher-risk for RTM effects.

Interpretation summary for Step 4a:

- Selections 1-3 are not likely to be affected substantially by RTM artifacts.
- Selection 4 has uncertain susceptibility to RTM artifacts (see Kramer 2016(6)).
- Selections 5-7 may exacerbate RTM artifacts and are higher-risk approaches.

<u>Step 4b</u>: Was the RTM effect quantified in relation to recovery/reversal frequencies? [For Type 3 growth variable, per Step 3]

Classify as follows:

1) **The frequency/incidence of reversal/recovery was analyzed in relation to a quantified RTM effect, which refers to the change in a subgroup due only to RTM.** To be classified as such, this requires a quantitative analysis (e.g., simulation).
This may have addressed RTM, but overall this approach based on a Type 3 variable (per Step 3) is sub-optimal.

2) ****No further analysis or simulation is performed to contextualize or interpret the reversal/recovery frequency.** This approach is higher-risk for RTM artifacts.

Note that classification in Step 4b as category 2 is correct even if:

i) the reversal/recovery variable is used in the downstream analysis (e.g., regression of reversal on a set of covariates), regardless of whether the baseline z-score is

included as a covariate in the regression model, because of sample selection using a baseline value (for example, HAZ below -2 = stunted).
ii) the authors commented in the text of the paper about the potential limitations of this analysis. In that situation, the mention of RTM should be captured by Part A, response 3 ('RTM is discussed as a factor that may affect or explain methods and/or results').

***Interpretation summary for Step 4b**:*

- Selection 1 may have addressed RTM, but the use of reversal/recovery classifications is inherently problematic, so this approach is not considered optimal even if the RTM effect is considered.
- Selection 2 signifies higher-risk of RTM artifacts.

***Step 5**: If the analysis is based on raw length or z-score data, rather than a derived child-level growth variable, classify the approach as follows:*

1) **Expression of a population-average summary (e.g., mean or median) or graphical depiction of the individual-level size data (e.g., Victora plot), for the population as a whole.** *This is very similar to what is commonly done in studies based on cross-sectional survey data*.

2) **Longitudinal modeling of repeated size measures** (e.g., mixed model, GEE). In this approach, the inferences are related to other variables (e.g., sex) that are associated with HAZ or height. Typically, child age is the longitudinal time scale. Baseline size is not distinguished from follow-up time points. There may be interactions between non-size variables of interest and age (e.g., sex-age interactions). In general, such approaches do not give rise to within-population RTM artifacts.

3) **Longitudinal modeling of repeated child-level size measures (e.g., mixed model, GEE) with adjustment for baseline size (such that baseline size is not included as one of the outcome values).** This is otherwise similar to classification 2 within this step (Step 5), and the adjustment for baseline size is not highly relevant to the consideration of RTM artifacts because the population-average size-by-age slope is unaffected by this adjustment.

4) **Other modeling approaches using raw height or HAZ data to create groups or patterns.** This should be selected for studies that primarily used modeling approaches that use longitudinal raw length or z-score data to create groups or patterns (e.g., mixture models, latent class analysis), whereby inferences are for the population as a whole and the model is used to estimate associations with non-growth variables (e.g., sex). Such approaches are not expected to give rise to within-population RTM artifacts, but this is uncertain.

5) ****Any of the above approaches stratified by baseline z-score.** This category supersedes any of the above-listed categories (1-4) whenever:
    - the trajectories, groups or patterns are stratified using a baseline HAZ/z-score OR
    - children are classified based on HAZ/z-score values (e.g. stunted or shortness (HAZ<-2), extremely stunted (HAZ<-3)) OR
    - any height-based categorization or depiction of categories or subgroups in graphical format is used whereby the rates of change in height/HAZ with age are allowed to differ across the sub-groups (e.g., study used different models for stunted vs non-stunted children)

    Irrespective of the specific modeling approach, the sub-grouping (or sub-sample selection) by baseline status can give rise to RTM artifacts driven by the mathematical coupling of baseline with subsequent change.

6) ****Longitudinal modeling of repeated child-level size measures (e.g., mixed model, GEE) with adjustment for an interaction term between age and baseline size.** This approach is very similar to #5 and is therefore at higher-risk of RTM artifacts for the same reasons. Note that this is distinct from the Conditional Random Slope approach (Leung et al. 2017) since the aim is to generate inferences about how baseline size modifies the rate/pattern of change in size with age (which amplifies RTM) rather than derive a RTM-corrected (conditional) measure at the individual child level.

Interpretation summary for Step 5*:*
- Approaches 1, 2 and 3 can be considered to either have addressed RTM, or RTM is not likely to be a major concern.
- Approach 4 is unlikely to give rise to RTM effects, but this is uncertain.
- *Approaches 5 and 6 may exacerbate RTM and are higher-risk approaches due to stratification by baseline values.*

Summary of Interpretations

Summary analysis is focused on the studies that used approaches that are higher-risk (versus lower risk) for RTM artifacts. The following higher-risk methods involve the mathematical coupling of baseline with subsequent change, or selection by baseline status:
- Step 4a: Selections 5-7
- Step 4b: Selection 2
- Step 5: Selections 5 and 6

Because the assessment of the methodological approach (Part B) is separate from whether and how RTM is mentioned in the paper (Part A), it is possible for a study to be considered in Part A as having explicitly intended to address RTM (type 4), yet be found in Part B to have used a method that was at high risk of RTM artifacts.

Supplementary Figure 1. Study selection flowchart

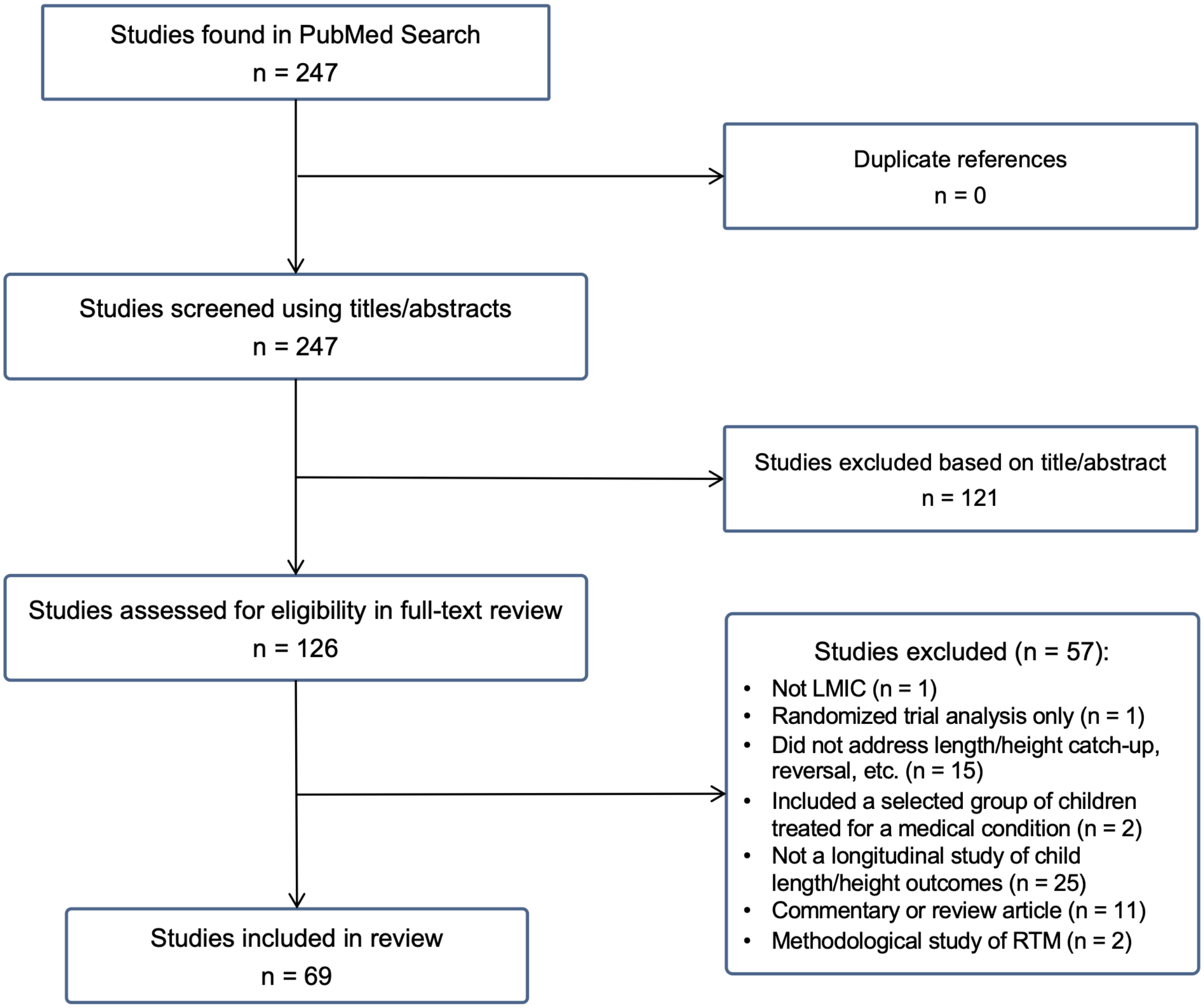

List of included studies

Study ID	Citation	**RTM mention classification** (See Supplementary Methods for categorization procedure)	**Model/analysis type** (see Supplementary Methods for categorization procedure and type definitions)	**RTM artifact risk assessment** (1=high-risk; 2=lower-risk)
Alisjahbana 2019	Alisjahbana et al. Intrauterine growth retardation (IUGR) as determinant and environment as modulator of infant mortality and morbidity: the Tanjungsari Cohort Study in Indonesia. Asia Pac J Clin Nutr 2019; 28(Suppl 1):S17-S31. doi: 10.6133/apjcn.201901_28(S1).0002.	1	5-5	1
Asiki 2019	Asiki et al. The effect of childhood stunting and wasting on adolescent cardiovascular diseases risk and educational achievement in rural Uganda: a retrospective cohort study. Glob Health Action 2019; 12(1):1626184. doi: 10.1080/16549716.2019.1626184.	1	4b-2	1
Batista 2012	Batista et al. Factors associated with height catch-up and catch-down growth among schoolchildren. PLoS One 2012; 7(3):e32903. doi: 10.1371/journal.pone.0032903.	1	4a-4	2
Belachew 2013	Belachew et al. Food insecurity and linear growth of adolescents in Jimma Zone, Southwest Ethiopia. Nutr J 2013; 12:55. doi: 10.1186/1475-2891-12-55.	1	5-3	2
Benjamin-Chung 2023	Benjamin-Chung et al. Early-childhood linear growth faltering in low- and middle-income countries. Nature 2023; 621(7979):550-557. doi: 10.1038/s41586-023-06418-5.	3	4b-2	1
Busert 2016	Busert et al. Dietary Diversity Is Positively Associated with Deviation from Expected Height in Rural Nepal. J Nutr 2016; 146(7):1387-93. doi: 10.3945/jn.115.220137.	1	3-4	2
Casale 2016	Casale et al. Recovery from stunting and cognitive outcomes in young children: evidence from the South African Birth to Twenty Cohort Study. J Dev Orig Health Dis 2016; 7(2):163-71. doi: 10.1017/S2040174415007175.	2	4b-2	1
Casale 2020	Casale et al. Catch-up growth in height and cognitive function: Why definitions matter. Econ Hum Biol 2020; 37:100853. doi: 10.1016/j.ehb.2020.100853.	1	4b-2	1
Checkley 2008	Checkley et al. Multi-country analysis of the effects of diarrhoea on childhood stunting. Int J Epidemiol 2008; 37(4):816-30. doi: 10.1093/ije/dyn099.	4	4b-1	2
Chhagan 2012	Chhagan et al. Clinical and contextual determinants of anthropometric failure at baseline and longitudinal improvements after starting antiretroviral treatment among South African children. Trop Med Int Health 2012; 17(9):1092-9. doi: 10.1111/j.1365-3156.2012.03026.x.	4	5-3	2
Coly 2006	Coly et al. Preschool stunting, adolescent migration, catch-up growth, and adult height in young senegalese men and women of rural origin. J Nutr 2006; 136(9):2412-20. doi: 10.1093/jn/136.9.2412.	4	4a-5	1
Crookston 2013	Crookston et al. Postinfancy growth, schooling, and cognitive achievement: Young Lives. Am J Clin Nutr 2013; 98(6):1555-63. doi: 10.3945/ajcn.113.067561.	1	3-4	2
Das 2022	Das et al. Do children in India grow well into adolescents? Longitudinal analysis of growth transitions from Young Lives panel survey in India. Public Health 2022; 202:18-25. doi: 10.1016/j.puhe.2021.10.010.	1	4b-2	1
Deichsel 2019	Deichsel et al. Birth size and early pneumonia predict linear growth among HIV-exposed uninfected infants. Matern Child Nutr 2019; 15(4):e12861. doi: 10.1111/mcn.12861.	3	5-2	2
Deng 2019	Deng et al. First-year growth of 834 preterm infants in a Chinese population: a single-center study. BMC Pediatr 2019; 19(1):403. doi: 10.1186/s12887-019-1752-8.	1	4a-4	2
Desmond 2017	Desmond et al. Catch-up growth in stunted children: Definitions and predictors. PLoS One 2017; 12(12):e0189135. doi: 10.1371/journal.pone.0189135.	3	4b-2	1
Duncan 2007	Duncan et al. Growth and development of children of mothers with leprosy and healthy controls. Ethiop Med J 2007; 45 Suppl 1:45558. doi: .	1	5-1	2
Ebissa 2016	Ebissa et al. Impact of highly active antiretroviral therapy on nutritional and immunologic status in HIV-infected children in the low-income country of Ethiopia. Nutrition 2016; 32(6):667-73. doi: 10.1016/j.nut.2015.12.035.	1	4a-2	2
Eckhardt 2005	Eckhardt et al. Growth patterns of Filipino children indicate potential compensatory growth. Ann Hum Biol 2005; 32(1):45365. doi: 10.1080/03014460400027607.	3	4a-5	1
Faye 2019	Faye et al. Factors associated with recovery from stunting among under-five children in two Nairobi informal settlements. PLoS One 2019; 14(4):e0215488. doi: 10.1371/journal.pone.0215488.	2	4b-2	1
Feinstein 2012	Feinstein et al. Effect of baseline immune suppression on growth recovery in HIV positive South African children receiving antiretroviral treatment. J Acquir Immune Defic Syndr 2012; 61(2):235-42. doi: 10.1097/QAI.0b013e3182634e09.	1	4b-2	1
Ferdous 2021	Ferdous et al. Early childhood malnutrition trajectory and lung function at preadolescence. Public Health Nutr 2021; 24(5):1009-1020. doi: 10.1017/S1368980019004853.	1	4a-4	2
Fink 2014	Fink et al. Childhood growth, schooling, and cognitive development: further evidence from the Young Lives study. Am J Clin Nutr 2014; 100(1):182-8. doi: 10.3945/ajcn.113.080960.	1	3-4	2
Friedman 2005	Friedman et al. Progression of stunting and its predictors among school-aged children in western Kenya. Eur J Clin Nutr 2005; 59(8):914-22. doi: 10.1038/sj.ejcn.1602161.	1	5-3	2
Fuglestad 2016	Fuglestad et al. Micronutrient status and neurodevelopment in internationally adopted children. Acta Paediatr 2016; 105(2):e67-76. doi: 10.1111/apa.13234.	1	5-2	2
Gandhi 2011	Gandhi et al. Height gain during early childhood is an important predictor of schooling and mathematics ability outcomes. Acta Paediatr 2011; 100(8):1113-8. doi: 10.1111/j.1651-2227.2011.02254.x.	1	3-4	2
Georgiadis 2017	Georgiadis et al. Growth recovery and faltering through early adolescence in low- and middle-income countries: Determinants and implications for cognitive development. Soc Sci Med 2017; 179:81-90. doi: 10.1016/j.socscimed.2017.02.031.	1	4b-2	1
Goddard 2024	Goddard et al. Prevalence, Incidence, and Reversal Pattern of Childhood Stunting From Birth to Age 2 Years in Ethiopia. JAMA Netw Open 2024; 7(1):e2352856. doi: 10.1001/jamanetworkopen.2023.52856.	1	4b-2	1
Gsponer 2012	Gsponer et al. Variability of growth in children starting antiretroviral treatment in southern Africa. Pediatrics 2012; 130(4):e966-77. doi: 10.1542/peds.2011-3020.	1	5-6	1
Hu 2016	Hu et al. Growth of HIV-Infected Children in the Early Stage of Antiretroviral Treatment: A Retrospective Cohort Study in China. AIDS Patient Care STDS 2016; 30(8):365-70. doi: 10.1089/apc.2016.0131.	1	4b-2	1
Hung 2005	Hung et al. Nutritional status following malaria control in a Vietnamese ethnic minority commune. Eur J Clin Nutr 2005; 59(8):891-9. doi: 10.1038/sj.ejcn.1602156.	1	5-6	1
Huynh 2015	Huynh et al. Longitudinal growth and health outcomes in nutritionally at-risk children who received long-term nutritional intervention. J Hum Nutr Diet 2015; 28(6):623-35. doi: 10.1111/jhn.12306.	1	5-5	1

Jesson 2015	Jesson et al. Effect of Age at Antiretroviral Therapy Initiation on Catch-up Growth Within the First 24 Months Among HIV-infected Children in the IeDEA West African Pediatric Cohort. Pediatr Infect Dis J 2015; 34(7):e159-68. doi: 10.1097/INF.0000000000000734.	1	4b-2	1
Jesson 2017	Jesson et al. Evaluation of a Nutritional Support Intervention in Malnourished HIV-Infected Children in Bamako, Mali. J Acquir Immune Defic Syndr 2017; 76(2):149-157. doi: 10.1097/QAI.0000000000001484.	1	4b-2	1
Kalanda 2005	Kalanda et al. Catch-up growth in Malawian babies, a longitudinal study of normal and low birthweight babies born in a malarious endemic area. Early Hum Dev 2005; 81(10):841-50. doi: 10.1016/j.earlhumdev.2005.06.006.	3	5-5	1
Kaur 2023	Kaur et al. Longitudinal Growth and Undernutrition Burden Among Term Low Birth Weight Newborns Reared in Adverse Socioeconomic Conditions in Delhi. Indian Pediatr 2023; 60(11):899-907. doi: www.indianpediatrics.net/nov2023/899.pdf.	3	4b-2	1
Kekitiinwa 2008	Kekitiinwa et al. Differences in factors associated with initial growth, CD4, and viral load responses to ART in HIV-infected children in Kampala, Uganda, and the United Kingdom/Ireland. J Acquir Immune Defic Syndr 2008; 49(4):384-92. doi: 10.1097/QAI.0b013e31818cdef5.	1	4a-1	2
Khatun 2004	Khatun et al. BRAC initiative towards promoting gender and social equity in health: a longitudinal study of child growth in Matlab, Bangladesh. Public Health Nutr 2004; 7(8):1071-9. doi: 10.1079/PHN2004652.	1	4b-2	1
Krishna 2016	Krishna et al. Short- and long-run associations between birth weight and children's height. Econ Hum Biol 2016; 21:156-66. doi: 10.1016/j.ehb.2016.02.004.	1	5-2	2
Kumar 2019	Kumar et al. Longitudinal Growth of Extremely Low Birth Weight Infants at 5¬†years. Indian J Pediatr 2019; 86(12):1139-1141. doi: 10.1007/s12098-019-03085-1.	1	5-5	1
Lelijveld 2020	Lelijveld et al. Long-term outcomes for children with disability and severe acute malnutrition in Malawi. BMJ Glob Health 2020; 5(10):e654-62. doi: 10.1136/bmjgh-2020-002613.	1	4a-4	2
Lundeen 2014	Lundeen et al. Height-for-age z scores increase despite increasing height deficits among children in 5 developing countries. Am J Clin Nutr 2014; 100(3):821-5. doi: 10.3945/ajcn.114.084368.	1	4b-2	1
Lundeen 2014	Lundeen et al. Growth faltering and recovery in children aged 1-8 years in four low- and middle-income countries: Young Lives. Public Health Nutr 2014; 17(9):2131-7. doi: 10.1017/S1368980013003017.	1	4b-2	1
Mwambenu 2022	Mwambenu et al. Growth and the pubertal growth spurt in South African adolescents living with perinatally-acquired HIV infection. PLoS One 2022; 17(1):e0262816. doi: 10.1371/journal.pone.0262816.	1	5-3	2
Nabwera 2018	Nabwera et al. Hormonal Correlates and Predictors of Nutritional Recovery in Malnourished African Children. J Trop Pediatr 2018; 64(5):364-372. doi: 10.1093/tropej/fmx075.	1	4a-7	1
Namirembe 2022	Namirembe et al. Child stunting starts in utero: Growth trajectories and determinants in Ugandan infants. Matern Child Nutr 2022; 18(3):e13359. doi: 10.1111/mcn.13359.	1	4b-2	1
Nemerimana 2023	Nemerimana et al. Factors associated with recovery from stunting at 24 months of age among infants and young children enrolled in the Pediatric Development Clinic (PDC): A retrospective cohort study in rural Rwanda. PLoS One 2023; 18(7):e0283504. doi: 10.1371/journal.pone.0283504.	1	4b-2	1
Ngari 2018	Ngari et al. The impact of rickets on growth and morbidity during recovery among children with complicated severe acute malnutrition in Kenya: A cohort study. Matern Child Nutr 2018; 14(2):e12569. doi: 10.1111/mcn.12569.	3	5-2	2
Ngari 2019	Ngari et al. Linear growth following complicated severe malnutrition: 1-year follow-up cohort of Kenyan children. Arch Dis Child 2019; 104(3):229-235. doi: 10.1136/archdischild-2018-315641.	4	4a-1	2
Oduro 2024	Oduro et al. Utilizing a multi-stage transition model for analysing child stunting in two urban slum settlements of Nairobi: A longitudinal analysis, 2011-2014. PLoS One 2024; 19(2):e0272684. doi: 10.1371/journal.pone.0272684.	1	4b-2	1
Parchure 2015	Parchure et al. Growth Patterns of HIV Infected Indian Children in Response to ART: A Clinic Based Cohort Study. Indian J Pediatr 2015; 82(6):519-24. doi: 10.1007/s12098-014-1659-1.	1	5-6	1
Pradeilles 2019	Pradeilles et al. Factors associated with catch-up growth in early infancy in rural Pakistan: A longitudinal analysis of the women's work and nutrition study. Matern Child Nutr 2019; 15(2):e12733. doi: 10.1111/mcn.12733.	4	3-4	2
Proos 2009	Proos et al. Growth & development of Indian children adopted in Sweden. Indian J Med Res 2009; 130(5):646-50. doi: .	1	5-5	1
Richard 2013	Richard et al. Diarrhea in early childhood: short-term association with weight and long-term association with length. Am J Epidemiol 2013; 178(7):1129-38. doi: 10.1093/aje/kwt094.	1	5-3	2
Rieger 2015	Rieger et al. Child health, its dynamic interaction with nutrition and health memory--evidence from Senegal. Econ Hum Biol 2015; 16():135-45. doi: 10.1016/j.ehb.2014.03.001.	1	5-4	2
Sania 2015	Sania et al. The contribution of preterm birth and intrauterine growth restriction to childhood undernutrition in Tanzania. Matern Child Nutr 2015; 11(4):618-30. doi: 10.1111/mcn.12123.	1	5-6	1
Schott 2013	Schott et al. Periods of child growth up to age 8 years in Ethiopia, India, Peru and Vietnam: key distal household and community factors. Soc Sci Med 2013; 97:278-87. doi: 10.1016/j.socscimed.2013.05.016.	1	3-4	2
Schott 2019	Schott et al. The double burden of malnutrition among youth: Trajectories and inequalities in four emerging economies. Econ Hum Biol 2019; 34:80-91. doi: 10.1016/j.ehb.2019.05.009.	1	5-4	2
Seth 2018	Seth et al. Effect of Antiretroviral Therapy on Growth Parameters of Children With HIV Infection. Pediatr Infect Dis J 2018; 37(1):85-89. doi: 10.1097/INF.0000000000001719.	1	5-6	1
Singh 2017	Singh et al. Birth Size, Stunting and Recovery from Stunting in Andhra Pradesh, India: Evidence from the Young Lives Study. Matern Child Health J 2017; 21(3):492-508. doi: 10.1007/s10995-016-2132-8.	1	4b-2	1
Sofiatin 2019	Sofiatin et al. Maternal and environmental risk for faltered growth in the first 5 years for Tanjungsari children in West Java, Indonesia. Asia Pac J Clin Nutr 2019; 28(Suppl 1):S32-S42. doi: 10.6133/apjcn.201901_28(S1).0003.	1	5-3	2
Sokolovic 2014	Sokolovic et al. Catch-up growth does not associate with cognitive development in Indian school-age children. Eur J Clin Nutr 2014; 68(1):14-8. doi: 10.1038/ejcn.2013.208.	1	4b-2	1
Sutcliffe 2011	Sutcliffe et al. Weight and height z-scores improve after initiating ART among HIV-infected children in rural Zambia: a cohort study. BMC Infect Dis 2011; 11:54. doi: 10.1186/1471-2334-11-54.	1	5-5	1
Svefors 2020	Svefors et al. Stunting, recovery from stunting and puberty development in the MINIMat cohort, Bangladesh. Acta Paediatr 2020; 109(1):122-133. doi: 10.1111/apa.14929.	1	4b-2	1

Teivaanmäki 2015	Teivaanmäki et al. Transition between stunted and nonstunted status: both occur from birth to 15 years of age in Malawi children. Acta Paediatr 2015; 104(12):1278-85. doi: 10.1111/apa.13060.	1	4b-2	1
Thomas 2020	Thomas et al. Age-related differences in height gain with dairy protein and micronutrient supplements in Indian primary school children. Asia Pac J Clin Nutr 2020; 29(2):355-362. doi: 10.6133/apjcn.202007_29(2).0018.	1	5-5	1
Traisathit 2019	Traisathit et al. Impact of antiretroviral treatment on height evolution of HIV infected children. BMC Pediatr 2019; 19(1):287. doi: 10.1186/s12887-019-1663-8.	1	5-2	2
Workicho 2019	Workicho et al. Adolescent pregnancy and linear growth of infants: a birth cohort study in rural Ethiopia. Nutr J 2019; 18(1):22. doi: 10.1186/s12937-019-0448-0.	1	5-3	2
Zhang 2024	Zhang et al. Growth trajectory of full-term small-for-gestational-age infants: a 3-year longitudinal study in China. BMJ Paediatr Open 2024; 8(1):e002278. doi: 10.1136/bmjpo-2023-002278.	1	5-4	2

Simulation Methods

We conducted simulations with synthetic longitudinal cohorts using a similar approach as described in a prior study(7, 8). In all simulations on the HAZ scale (Figures 1, 2 and 3), we assumed a normal distribution of height-for-age z-scores (HAZ) at each age timepoint and used a fixed standard deviation (SD) of 1.0 for any specified mean HAZ, which is consistent with the assumption of a harmonic whole-population shifting of the HAZ distribution. In all simulations, the correlation (r) between values at any two age timepoints were based on a correlation matrix derived from data from the placebo group of a Bangladeshi trial cohort(9). All simulations included 100,000 participants, and we assumed no missingness so there were 100,000 observations at each age. We used the *drawnorm* command in Stata to generate the correlated distributions at each age timepoint based on the specified means, standard deviations, and correlation matrices.

To generate Figure 1, a cohort was simulated that was observed at 3, 6, 12, 18 and 24 months of age. In one scenario, there was a stable mean HAZ=-1 at all ages; in a second scenario, there was whole-population linear growth faltering pattern whereby the mean HAZ declined by 0.25 at each successive age timepoint. Upward threshold-crossing events were defined when a stunted child (HAZ<-2) at one age timepoint was no longer stunted (HAZ>=-2) at a subsequent age timepoint, which corresponds to 'stunting reversal' or 'recovery from stunting' in some published studies in the literature.

Figure 2 was generated using a similar simulation approach as Figure 1, but with a fixed mean HAZ=0. Downward and upward threshold-crossing events were defined using thresholds of +1 and -1, respectively, which were selected for illustration purposes to show a higher proportion of crossing events than would occur across thresholds of +2 and -2, given the mean HAZ=0.

Figure 3 was generated for a cohort only observed at two age timepoints (3 and 24 months of age) with stable mean HAZ=-1, but was otherwise similar to the simulations used for Figures 1 and 2. The correlation (*r*) between HAZ and 3 months and 24 months was 0.75. Upward threshold-crossing was assessed in two subgroups of stunted children distinguished by their HAZ values at 3 months of age: HAZ<-2 and HAZ≥-2.5 versus HAZ<-2.5. Upward threshold-crossing events were defined when a child who was stunted at 3 months was no longer stunted (HAZ>=-2) at 24 months. We also calculated the change in mean HAZ (mean ∆HAZ) and standard deviation (SD) for the mean ∆HAZ from 3 to 24 months of age for each stunted subgroup.

Figure 4 was generated using a simulation that was analogous to that used for Figure 3, but on the raw length scale. As in the Figure 3 simulation, the synthetic cohort was only observed at two age timepoints (3 and 24 months of age). The distributions at both ages corresponded to a mean HAZ = –1 and standard deviation (SD) based on the World Health Organization Child Growth Standards (WHO-GS) for girls using the length-for-age standards from birth to 2 years (https://www.who.int/tools/child-growth-standards/standards/length-height-for-age): at 3 months, mean = 57.7 cm (SD 2.1); at 24 months, mean = 83.2 cm (SD 3.2). As in Figure 3, we set the correlation between lengths at 3 months and 24 months to *r* = 0.75. Height-for-age difference (HAD) values were calculated by subtracting each individual child length from the WHO-GS median for

girls: at 3 months, median = 59.8 cm; at 24 months, median = 86.4 cm. The term HAD is conventionally used in the literature to refer to differences from the median based on either length-for-age or height-for-age standards. Upward threshold-crossing was assessed in two subgroups of relatively shorter children distinguished by their lengths at 3 months of age: one group was defined by length less than the 10th percentile and above the 5th percentile at 3 months; the other group was defined by length less than the 5th percentile at 3. On the original raw length scale (panel A), catch-up was defined when a child in one of these groups was above the empirical 10th percentile of the population at 24 months. The above-mentioned percentiles were empirically defined for the simulated population and thus do not correspond to percentiles of the WHO-GS. The 10th-percentile threshold for catch-up was selected arbitrarily to illustrate changes in subgroups selected from the lower tail of the baseline distribution, relative to the rest of the simulated population. On the HAD scale (panel B), catch-up was defined as occurring when HAD at 24 months was of lower magnitude than at 3 months (i.e., ΔHAD>0, such that HAD is closer to 0 at 24 months than at 3 months). For each subgroup, we calculated the change in mean body length (Δlength) and standard deviation (SD) for the mean Δlength, change in mean HAD (ΔHAD) and standard deviation (SD) for the mean ΔHAD, and proportions of children with of 'catch-up' as defined above for Δlength and ΔHAD from 3 to 24 months of age.

For all figures, we used random 0.5% subsamples of observations to aid in visualizing threshold crossing event frequences at each age.

All simulations were implemented and figures generated using Stata 16.1 (StataCorp, College Station, TX)(10). We used a formula published by Linden et al. and implemented using the STATA package *rtmci* to estimate the RTM effect(11), to produce results described in the legend for Figure 1.

The parameters used to simulate all datasets and complete correlation matrices used in the simulated datasets are publicly available online: https://github.com/DGBassaniLab/LGF_RTM.

References

1. Leroy JL, Ruel M, Habicht J-P, Frongillo EA. Using height-for-age differences (HAD) instead of height-for-age z-scores (HAZ) for the meaningful measurement of population-level catch-up in linear growth in children less than 5 years of age. BMC Pediatr. 2015;15(1):145. doi: 10.1186/s12887-015-0458-9.

2. Karlsson O, Kim R, Moloney GM, Hasman A, Subramanian SV. Patterns in child stunting by age: A cross-sectional study of 94 low- and middle-income countries. Matern Child Nutr. 2023;19(4):e13537. Epub 20230605. doi: 10.1111/mcn.13537. PubMed PMID: 37276243; PubMed Central PMCID: PMC10483943.

3. Cameron N, Preece MA, Cole TJ. Catch-up growth or regression to the mean? Recovery from stunting revisited. Am J Hum Biol. 2005;17(4):412-7. doi: 10.1002/ajhb.20408. PubMed PMID: 15981181.

4. Leung M, Bassani DG, Racine-Poon A, Goldenberg A, Ali SA, Kang G, et al. Conditional random slope: A new approach for estimating individual child growth velocity in epidemiological research. Am J Hum Biol. 2017;29(5). Epub 2017/04/22. doi: 10.1002/ajhb.23009. PubMed PMID: 28429467; PubMed Central PMCID: PMC5599979.

5. Covidence systematic review software, Veritas Health Innovation, Melbourne, Australia. Available at www.covidence.org.

6. Kramer MS, Zhang X, Bin Aris I, Dahhou M, Naimi A, Yang S, et al. Methodological challenges in studying the causal determinants of child growth. Int J Epidemiol. 2016;45(6):2030-7. doi: 10.1093/ije/dyw090. PubMed PMID: 27297676.

7. Roth DE, Watson KM, Bassani DG. Stunting incidence and reversal as metrics of postnatal linear growth faltering in low- and middle-income countries: a critical appraisal and simulation study. BMJ Open. 2025;15(9):e097286. Epub 20250928. doi: 10.1136/bmjopen-2024-097286. PubMed PMID: 41022435; PubMed Central PMCID: PMC12481314.

8. Roth DE, Watson KM, Bassani DG. Whole-population perspective is needed for analyses and actions to address linear growth faltering in low- and middle-income countries. medRxiv [Internet]. 2024. Available from: http://medrxiv.org/content/early/2024/06/25/2024.06.24.24309409.abstract.

9. Roth DE, Morris SK, Zlotkin S, Gernand AD, Ahmed T, Shanta SS, et al. Vitamin D Supplementation in Pregnancy and Lactation and Infant Growth. The New England Journal of Medicine. 2018;379(6):535-46. doi: 10.1056/NEJMoa1800927. PubMed PMID: 30089075; PubMed Central PMCID: PMC6004541.

10. StataCorp. 2020. Stata Statistical Software: Release 16.1. College Station, TX: StataCorp LLC.

11. Linden A. Assessing regression to the mean effects in health care initiatives. BMC medical research methodology. 2013;13:119. Epub 20130928. doi: 10.1186/1471-2288-13-119. PubMed PMID: 24073634; PubMed Central PMCID: PMC3849564.
